# Advances and Challenges in Non-Contact Acoustic Signal-Based Bearing Fault Diagnosis: A Review

**DOI:** 10.3390/s26175638

**Published:** 2026-09-04

**Authors:** Shengkai Zhao, Hongjie Cheng, Yuan Zhao, Binqiang Wang, Zhen Huang

**Affiliations:** School of Missile Engineering, Rocket Force University of Engineering, Xi’an 710025, China

**Keywords:** fault diagnosis, bearings, deep learning, acoustic signals, multi-modal fusion, condition monitoring

## Abstract

Bearings are core components of rotating machinery, and the development of precise and efficient fault diagnosis technologies is of paramount importance for realizing early warning and accurate localization of faults. This paper briefly analyzes bearing fault mechanisms and provides a comprehensive review and critical commentary on the research progress of bearing fault diagnosis methods, outlining future development trends. Specifically, this study begins by introducing common bearing fault types and reviewing the advancements in fault signal acquisition techniques. Subsequently, it categorizes fault diagnosis methods based on vibration signals and critically evaluates their respective research methodologies. Furthermore, focusing on non-contact acoustic signal diagnosis, the paper summarizes mainstream technical pathways for acoustic signal denoising and highlights innovative applications of deep-learning models tailored to acoustic characteristics. Finally, addressing the urgent demands for industrial deployment, future research directions are projected from three perspectives: the deep integration of physics-driven and data-driven multi-modal fusion, interpretable diagnosis assisted by Large Language Models (LLMs), and lightweight engineering deployment. This work aims to provide a reference for constructing an all-scenario intelligent monitoring system.

## 1. Introduction

Bearings are core foundational components of modern industrial equipment and rotating machinery, primarily fulfilling critical functions such as supporting rotating shafts, minimizing frictional losses, guaranteeing rotational precision, and withstanding complex alternating loads. They possess irreplaceable application value in numerous high-end equipment fields, including rail transit, aerospace, wind power energy, and precision manufacturing [1,2]. With the continuous advancement of industrial intelligence and equipment automation, bearings in various core devices are frequently subjected to harsh operating conditions characterized by high speeds, heavy loads, and variable working conditions. This is compounded by the coupled effects of multiple random factors, such as environmental disturbances, material fatigue, and assembly errors [3]. Consequently, potential faults during operation are difficult to eliminate entirely. Moreover, the health status of bearings directly determines the operational stability, machining accuracy, and safety production levels of the entire system. As the primary cause of rotating machinery failures, bearing faults account for over 40% of related equipment failures [4]. Therefore, conducting precise early-stage diagnosis of bearing faults constitutes a core technological imperative for realizing full lifecycle health monitoring of equipment, ensuring continuous and reliable operation, and advancing the implementation of predictive maintenance. This endeavor holds significant engineering application value and theoretical research significance [5,6,7].

**Figure 1:**
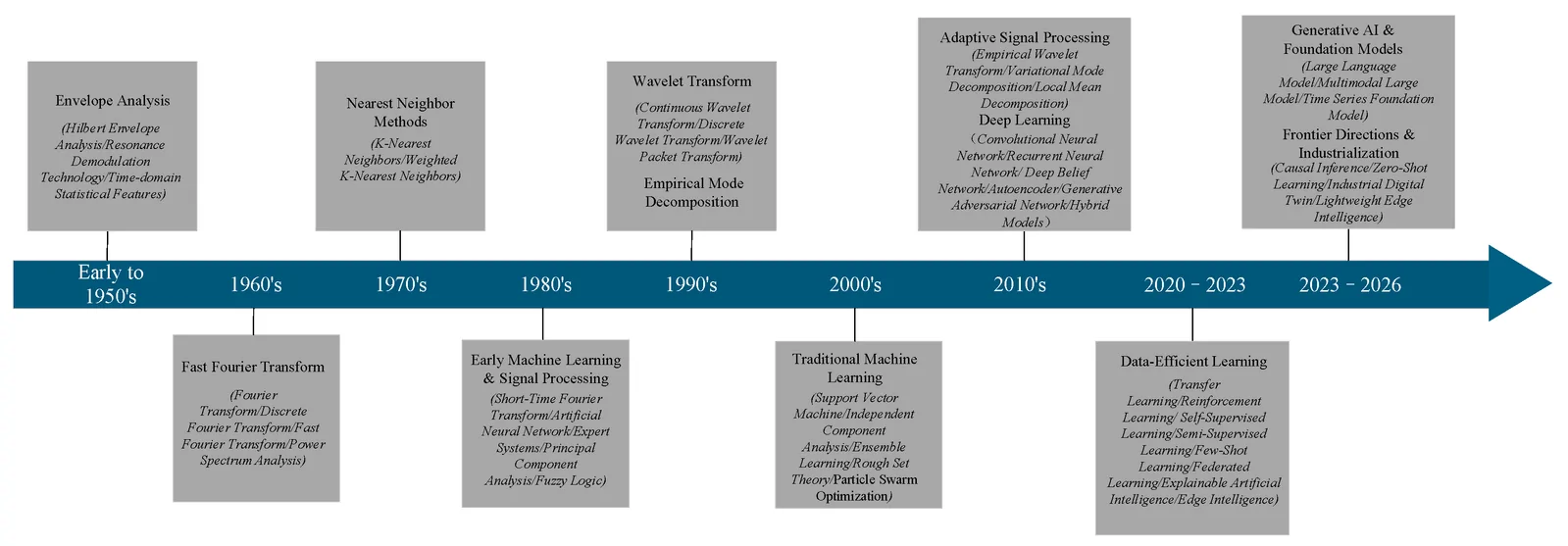
Evolution of bearing fault diagnosis technology. The timeline entries correspond to the seminal references cited in the text [17,18,19,20,21,22,23,24,25,26,27,28,29,30,31].

The identifiability of bearing faults provides the foundation for the development and iteration of diagnostic technologies. As illustrated in Figure 1, scholars worldwide have conducted extensive and in-depth investigations into bearing fault diagnosis, yielding numerous research achievements with substantial theoretical depth and engineering value. The chronological entries in Figure 1 follow the three-dimensional taxonomy introduced in Section 4.2, namely learning paradigms, model architectures, and transfer strategies, so that no architecture is assigned to a single learning paradigm. Currently, a multi-dimensional monitoring system for bearing fault diagnosis has been established, with mainstream methodologies encompassing feature extraction and pattern recognition techniques based on diverse measurement modalities, including vibration signals [8,9,10], temperature fields [11,12], infrared thermography [13], and acoustic signals. Among these, vibration signals are the most widely adopted and technologically mature means in fault diagnosis due to their strong correlation with fault characteristics. However, this method relies on contact-based sensor deployment and faces technical limitations in special scenarios, such as rotating bearing components, including restricted sensor installation, vulnerability to impact damage, and limited monitoring coverage [14]. Acoustic signals enable non-contact acquisition and can capture early impulsive faults that are difficult for vibration-based methods to identify, yet they are susceptible to environmental noise interference and feature aliasing [15]. Temperature signals permit non-contact monitoring but exhibit low sensitivity to early, incipient internal faults, with insignificant changes in signal characteristics. Infrared thermography also offers non-contact detection advantages but is constrained by high hardware and deployment costs, hindering its large-scale adoption in industrial settings [16]. In contrast, fault diagnosis based on single signals has become increasingly inadequate in meeting the escalating complexity of modern industrial environments. Consequently, multi-modal signal fusion diagnosis is imperative to address susceptibility to interference and the fragmented nature of features inherent in single-signal approaches, thereby substantially enhancing the reliability and robustness of bearing fault diagnosis under complex operating conditions. Following decades of iterative development, bearing fault diagnosis technology has exhibited overarching evolutionary trends: a shift from reliance on manual feature engineering to adaptive feature learning, from local feature perception to global correlation modeling, and from fully labeled ideal laboratory scenarios to adaptation for real-world industrial settings with scarce labels.

The continuous evolution of bearing fault diagnosis technology has provided critical support for the safe operation of industrial equipment. In particular, the rapid advancement of deep learning has further propelled research and application of bearing fault diagnosis based on non-vibration signals. Existing review articles predominantly concentrate on the theoretical progress of vibration signals and deep-learning methodologies while lacking a systematic synthesis of non-contact monitoring technologies such as acoustic signals and failing to provide an in-depth analysis of the gap between laboratory algorithms and industrial deployment. Motivated by this, this paper focuses on the tasks of bearing fault detection and classification, presenting a critical review. Firstly, it outlines the mechanisms of common bearing fault types, summarizes the fundamental workflow of bearing fault diagnosis, and critically evaluates the principles, advantages, limitations, and applications of classical traditional methods in the field. Secondly, considering the complexity, dynamics, and nonlinearity of operational data, it highlights the unique advantages and immense potential of deep-learning techniques in the stages of fault feature extraction and pattern recognition. Thirdly, it elaborates on the principles, merits, drawbacks, and applications of current representative deep-learning approaches while supplementing recent research progress in acoustic signal-based bearing fault diagnosis. Finally, aligning with the practical requirements and current research status of bearing fault diagnosis, it identifies key technical challenges that urgently need to be addressed, and it provides an outlook on future research directions and development trends, aiming to offer references and insights for subsequent related studies.

This review is presented as a critical (narrative) review rather than a formal systematic review; accordingly, the literature collection described below follows a structured but non-exhaustive procedure. The literature search was conducted in August 2026 across the Web of Science Core Collection, Scopus, IEEE Xplore, and Google Scholar, supplemented by CNKI for Chinese-language studies. No lower time bound was imposed so that seminal works could be retained, while systematic emphasis was placed on studies published between 2015 and 2026, with the most recent developments covered up to August 2026. Three keyword groups were combined with Boolean operators: (i) domain terms, including “bearing fault diagnosis”, “rolling bearing”, “bearing failure”, and “rotating machinery fault”; (ii) signal-modality terms, including “acoustic signal”, “airborne sound”, “acoustic emission”, “microphone array”, and “vibration signal”; and (iii) method terms, including “deep learning”, “machine learning”, “convolutional neural network”, “transformer”, “large language model”, “signal processing”, “multi-modal fusion”, and “transfer learning”. Title, abstract, and keyword fields were searched, and backward and forward citation tracking of highly relevant articles was performed to complement the keyword search, and (iv) a small number of closely related rotating-machinery studies, such as those on gears or pumps, whose methodological frameworks transfer directly to bearing diagnosis and are explicitly marked as non-bearing cases in the text.

Inclusion and exclusion criteria. The inclusion criteria were: (i) peer-reviewed journal articles and conference papers; (ii) studies addressing bearing fault detection, classification, localization, or remaining useful life prediction using acoustic or vibration signals; and (iii) methodological studies on signal denoising, feature extraction, or learning-based diagnosis relevant to the above tasks. The exclusion criteria were: (i) non-peer-reviewed sources such as technical blogs and unpublished reports; (ii) duplicate records and studies not focused on bearing faults, such as pure tribology or lubrication chemistry; (iii) studies whose full text was inaccessible; and (iv) publications in languages other than English and Chinese.

Screening process and outcome. The retrieved records were screened based on their titles, abstracts, and full texts. After screening, 162 references were finally included, of which approximately 75% were published from 2020–2026 and about 48% from 2024–2026, reflecting the fast-moving nature of this field. The included studies were organized into the thematic structure of this review.

## 2. Bearing Fault Mechanisms and Types

**Figure 2:**
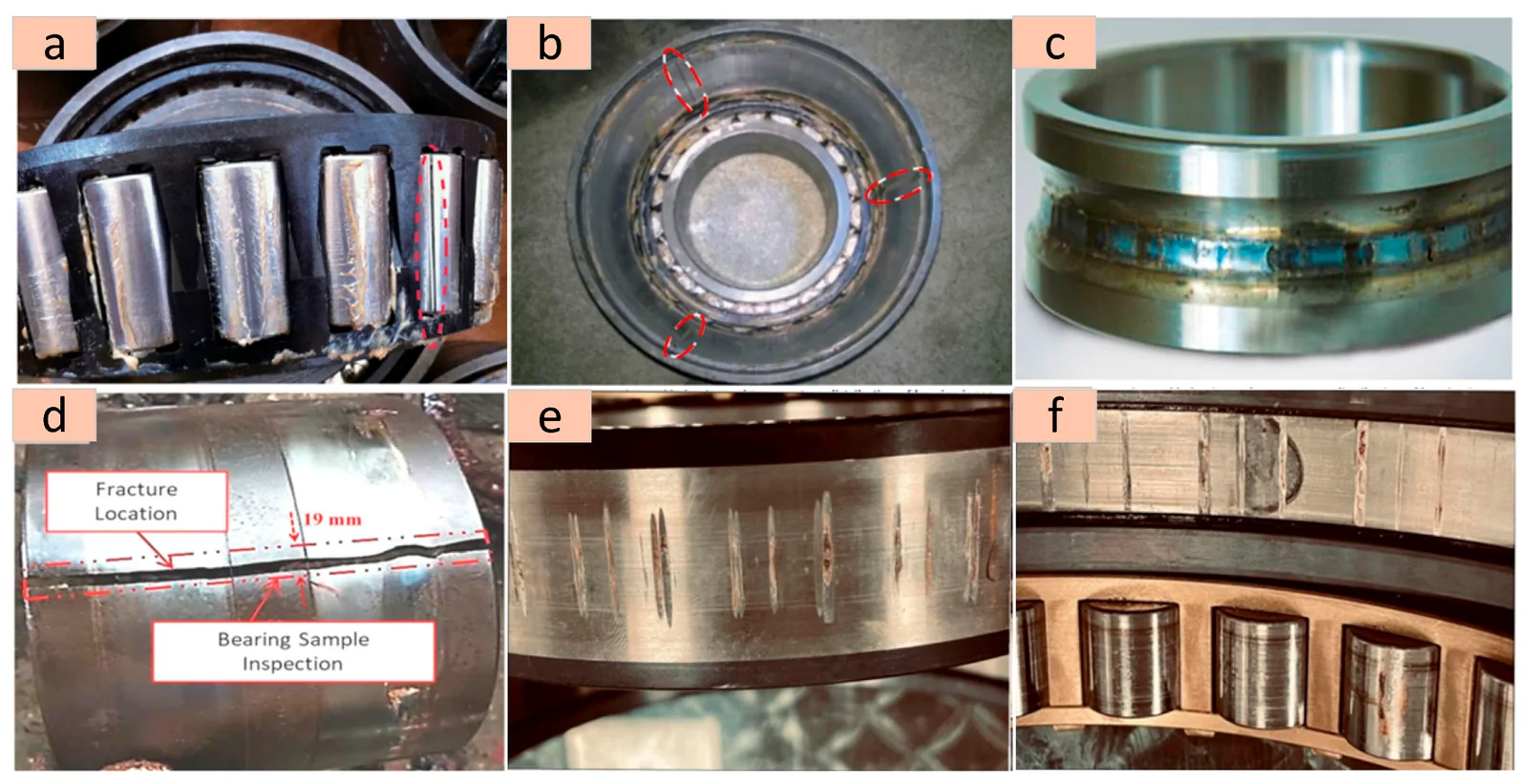
Typical Fault Types of Bearings: (**a**) Roller wear; (**b**) Outer ring failure; (**c**) Overheating of bearing; (**d**) The fracture on bearing; (**e**) False brinelling on the bearing surfaces; (**f**) Fatigue spalling of the Inner raceway. Reproduced with permission from [45]. Copyright 2025, ASM International, published by Springer.

Bearing fault types are intrinsically linked to their core components and operating conditions. Taking the most widely used rolling bearings as an example, faults are primarily associated with four key components: the inner race, outer race, rolling elements, and cage [32]. As illustrated in Figure 2, typical failure modes can be categorized into two classes. The first class comprises faults directly related to core components, such as fatigue spalling [33], wear [34], corrosion [35], electrical erosion [36], plastic deformation [37], and cracking or fracture [38]. The second class includes auxiliary faults like fit failure [39], lubrication failure [40], contamination failure [41], and overheating failure [42]. Research indicates that bearing faults stem not only from structural damage to the cage, rolling elements, and rings but also from the combined effects of operational environmental factors, including deteriorated lubrication, installation errors, and foreign object ingress [43].

Based on the distribution characteristics of defects, bearing faults can be further classified into localized defects and distributed defects. Localized defects include pitting, dents, and cracks, while distributed defects involve abnormal surface roughness and dimensional errors in rolling elements. The vast majority of the aforementioned faults are identifiable. By collecting corresponding signals via sensors such as vibration, acoustic emission, temperature, and current [44], and applying signal processing and intelligent diagnostic algorithms, various bearing faults can be effectively identified. This process allows for the accurate determination of fault locations and severity. Among these, localized defects in the inner race, outer race, and rolling elements represent the most recognizable fault types. These faults currently offer the highest diagnostic accuracy and the most mature application. In contrast, distributed defects induce overall changes in signal energy, rendering them comparatively more challenging to detect.

Furthermore, various failure modes in bearings are not independent; they exhibit significant coupled induction and cascading evolution effects during actual service. A thorough understanding of bearing failure mechanisms clarifies the physical essence and characteristic mapping relationships throughout the fault lifecycle. This not only provides theoretical support for the entire workflow of bearing design, manufacturing, and operation and maintenance but also constitutes the core foundation for constructing a high-precision fault diagnosis system.

## 3. Bearing Fault Diagnosis Process and Methodologies

**Figure 3:**
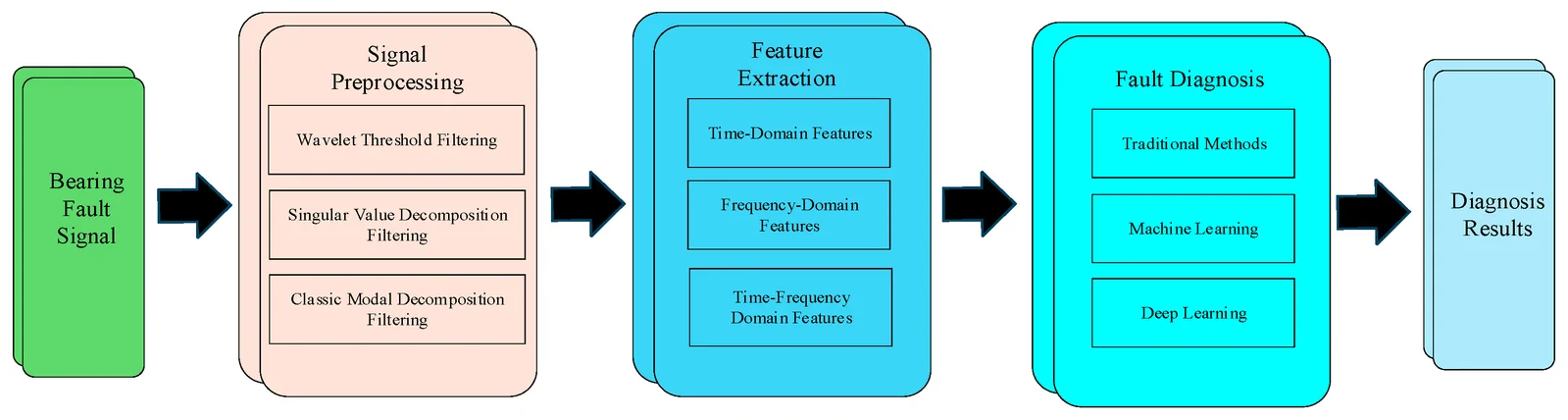
Diagnostic flowchart.

In practical industrial settings, bearings are frequently subjected to harsh operating environments characterized by prolonged high-speed rotation and complex coupled alternating loads. The initiation and progression of faults under such conditions exhibit a high degree of concealment, necessitating the implementation of routine condition monitoring to precisely track their health evolution patterns. Figure 3 illustrates the core technical workflow of bearing fault diagnosis. Initially, raw bearing signals are acquired and preprocessed. Noise reduction algorithms, such as wavelet threshold filtering and singular value decomposition filtering, are employed to purify the signals and enhance the signal-to-noise ratio (SNR). Subsequently, multidimensional feature parameters in the time, frequency, and time-frequency domains are further extracted and mined. Following this, traditional pattern recognition methods or deep-learning models are utilized to accomplish fault pattern classification and localization. Ultimately, precise and reliable fault diagnosis conclusions are outputted.

### 3.1. Signal Acquisition and Preprocessing

Signal acquisition constitutes the foundational stage of bearing fault diagnosis. The selection of acquisition hardware directly governs the accuracy of subsequent fault feature extraction and diagnostic outcomes; therefore, appropriate sensors must be chosen based on signal modalities and specific diagnostic requirements. Currently, core sensors for vibration signal acquisition in the fault diagnosis field include accelerometers compatible with a wide frequency range (piezoelectric [46], Micro-Electro-Mechanical System (MEMS) [47]), velocity sensors suitable for low-frequency vibration scenarios (electromagnetic [48]), and displacement sensors for high-precision diagnostics (eddy current, capacitive). In certain scenarios, Acoustic Emission (AE) sensors [49] are additionally deployed to capture the elastic waves accompanying vibration, thereby assisting in early fault identification. A comprehensive survey of non-destructive sensing techniques for wind turbine condition and structural health monitoring, covering acoustic emission, ultrasonic, thermographic, and electromagnetic methods across more than 300 works, is provided by Civera and Surace [50], which positions the sensor discussion of this section within the broader landscape of non-destructive testing.

For non-vibration signals, sensors dedicated to acoustic signal acquisition encompass single-channel microphones, microphone arrays [51], and acoustic cameras [52]. Sensors for current signal acquisition include Rogowski coils and Hall-effect current sensors [53]. The measurement characteristics of these diverse signals differ significantly. Vibration signals offer high measurement precision but necessitate contact-based installation. Acoustic signals permit non-contact measurement and convenient deployment but are susceptible to environmental noise interference, resulting in a low signal-to-noise ratio. Current signals incur the lowest measurement cost but suffer from insufficient sensitivity. Infrared signals exhibit delayed measurement response and are exclusively suitable for monitoring overheating faults.

#### Signal Preprocessing

In industrial environments, acquired signals are ubiquitously contaminated by environmental noise and redundant components, so preprocessing is imperative to enhance the discernibility of fault characteristics. Commonly employed methodologies primarily encompass classical filtering techniques such as adaptive filtering [54], Singular Value Decomposition (SVD) filtering, Wavelet Transform (WT) filtering, and Empirical Mode Decomposition filtering. Furthermore, the repertoire includes stochastic resonance, chaotic oscillators [55], and differential oscillators [56], as well as integrated filtering schemes combining adaptive Variational Mode Decomposition (VMD) [57] and adaptive wavelet threshold denoising [58,59].

### 3.2. Feature Extraction

Feature extraction constitutes a pivotal procedure within the bearing fault diagnosis workflow. Its core objective is to mine and extract characteristic parameters from preprocessed signals. Based on the dimensionality of the extracted features, commonly utilized signal feature extraction methodologies are primarily categorized into three classes. These include time-domain feature extraction, frequency-domain feature extraction, and time-frequency domain feature extraction, as detailed below.

**Figure 4:**
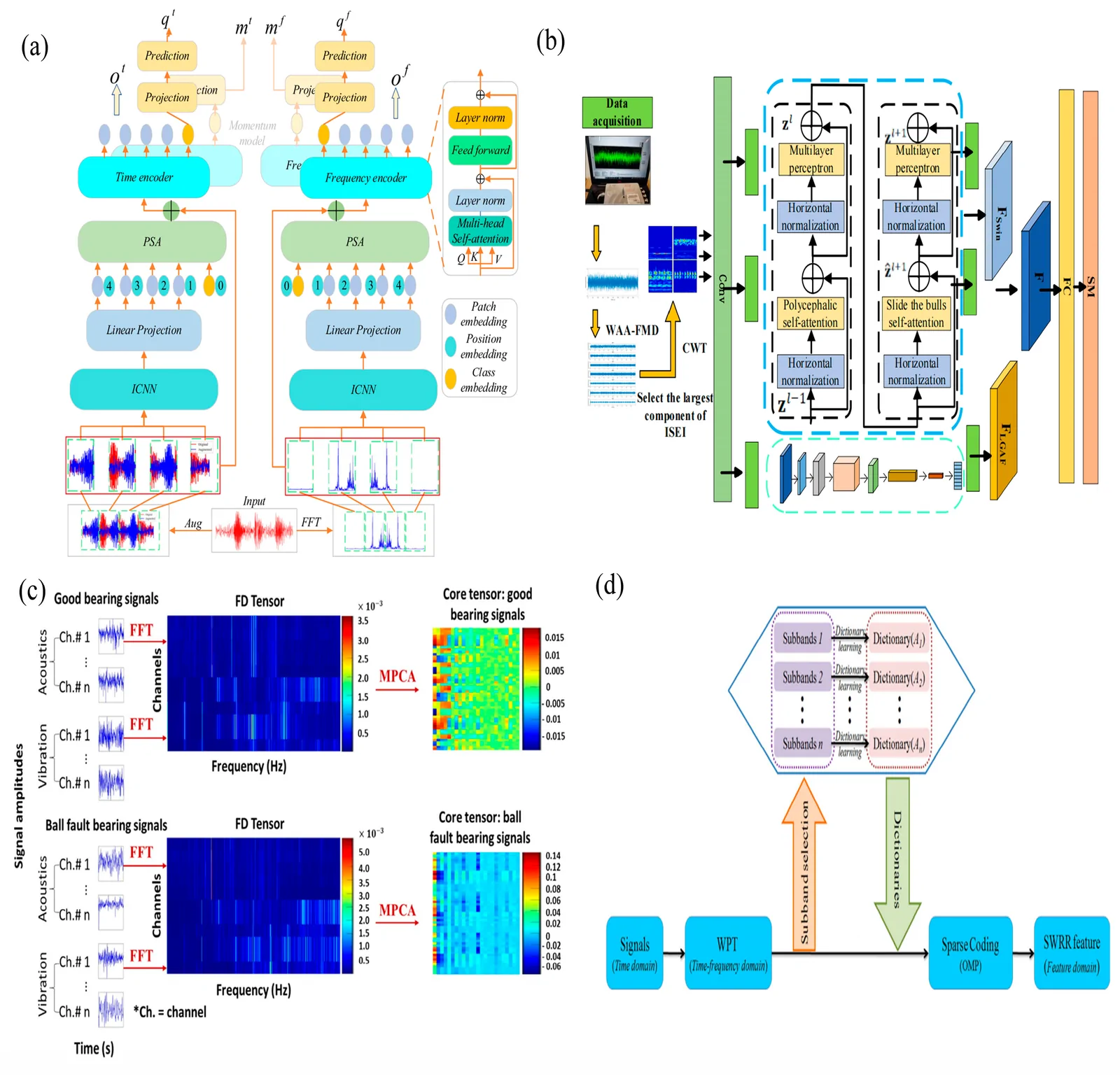
Time-frequency feature extraction and fusion for bearing fault diagnosis. (**a**) Structure and workflow of dual encoder, reproduced with permission from [61], copyright 2025, Springer; (**b**) Fault Diagnosis Model of LGAF-Swin Transformer, reproduced from [63] under CC BY 4.0; (**c**) Frequency domain–based tensor from original time domain signals, reproduced with permission from [65], copyright 2022, Springer; (**d**) Procedure of SWRR feature extraction method, reproduced with permission from [69], copyright 2015, Springer.

The core logic of time-domain analysis resides in leveraging the variation patterns of signal amplitude over time. Statistical parameters such as mean, variance, kurtosis, and peak factor are utilized to characterize the anomalous responses induced by faults. Among these, kurtosis is regarded as a core indicator for early fault detection due to its extreme sensitivity to transient impulses. Although computationally simple, these methods have weak anti-noise performance and suit low-noise surface-damage diagnosis. To address these limitations, the academic community has reinforced these methods through noise-robust restoration and lightweight adaptation. For instance, Li et al. [60], on acoustic emission signals, introduced autocorrelation functions to suppress background noise and incorporated an improved Generalized Multipoint Optimal Minimum Entropy Deconvolution Adjusted method to enhance impact components. Regarding engineering deployment, as illustrated in Figure 4a, He et al. constructed a dedicated time-domain encoder for scenarios with limited labels [61]. Samanta et al. selected five typical features, including root mean square and skewness, and integrated them with neural networks [62], effectively capitalizing on the computational efficiency of time-domain methods. Nevertheless, time-domain analysis remains fundamentally a description of superficial signal amplitude. It struggles to independently capture complex fault patterns and must therefore be employed in conjunction with frequency-domain analysis, time-frequency analysis, or deep-learning models.

The core logic of frequency-domain analysis lies in leveraging the Fourier Transform to map time-domain signals into the frequency domain. By capturing the physical signature of fault characteristic frequencies and their harmonics for localization and identification. However, constrained by the global integration nature of the Fourier Transform, it fails to characterize the time-varying properties of frequency components under engineering conditions involving variable speeds and loads. In response, targeted optimizations have been proposed in the literature. For instance, addressing the issue of weak fault features being masked by strong background noise, Wang et al. [63] introduced the Hilbert Transform to extract the analytic signal envelope, accurately pinpointing fault frequencies via envelope spectrum analysis, as illustrated in Figure 4b. Beyond classical spectral parameters, entropy-based spectral features have demonstrated value on real field data: Civera et al. [64] combined instantaneous spectral entropy with the continuous wavelet transform to detect the bearing failure of an operating wind turbine, reporting a computationally efficient pipeline suitable for continuous condition monitoring. To tackle the limitation of single-feature representation, Al Mamun et al. [65] utilized the Fast Fourier Transform (FFT) to synchronously convert multi-source acoustic and vibration signals into the frequency domain, constructing a multi-dimensional feature tensor subsequently reduced via Multilinear Principal Component Analysis. This approach provides robust support for deep multi-sensor fusion, as depicted in Figure 4c. Furthermore, to enhance adaptive capability, Agah et al. [66] combined Variational Mode Decomposition with Power Spectral Density (PSD) analysis, filtering effective Intrinsic Mode Functions (IMFs) prior to spectral analysis, which significantly improved frequency localization accuracy under complex operating conditions. While frequency-domain analysis demonstrates exceptional performance under stationary signal conditions, its inherent deficiency in handling non-stationary signals remains irremediable. This compels researchers to transcend the bottleneck of single-frequency perspectives and pursue joint time-frequency analysis frameworks.

Time-frequency analysis aims to address the inability of traditional frequency-domain methods to process non-stationary signals. By synchronously extracting two-dimensional features from both the time and frequency domains, it precisely characterizes the dynamic evolution patterns of fault frequencies under varying operating conditions. However, Time-frequency methods remain constrained by the resolution trade-off (STFT is limited by the Heisenberg uncertainty principle) and by basis-function dependence (WT requires predefined bases). To overcome these limitations, Lourari et al. [67] employed the Hilbert-Huang Transform (HHT), while Sun et al. [68] utilized Complementary Ensemble Empirical Mode Decomposition for adaptive time-frequency representation, attempting to circumvent the constraints of preset basis functions. Concurrently, as illustrated in Figure 4d, Wang et al. [69] integrated the Wavelet Packet Transform (WPT) with sparse representation theory, and Gao et al. [70] applied Non-negative Matrix Factorization (NMF) to reduce dimensionality and cluster STFT features, thereby enhancing fault-sensitive frequency bands and suppressing redundant information. Although time-frequency analysis offers significant advantages for non-stationary signal diagnosis, inherent risks of mode mixing and the complexity of manual feature design still limit its generalization capability under extreme operating conditions, indicating room for optimization in engineering applications.

## 4. Fault Diagnosis Methods Based on Bearing Vibration Signals

### 4.1. Fault Diagnosis Methods Based on Traditional Machine Learning

While feature extraction techniques can capture relevant indicators of bearing faults, accurate fault classification necessitates further effective categorization. In the field of fault diagnosis, conventional approaches predominantly employ data-driven machine-learning methods [71]. These algorithms analyze and discriminate extracted fault features, with typical applications summarized as follows.

**Figure 5:**
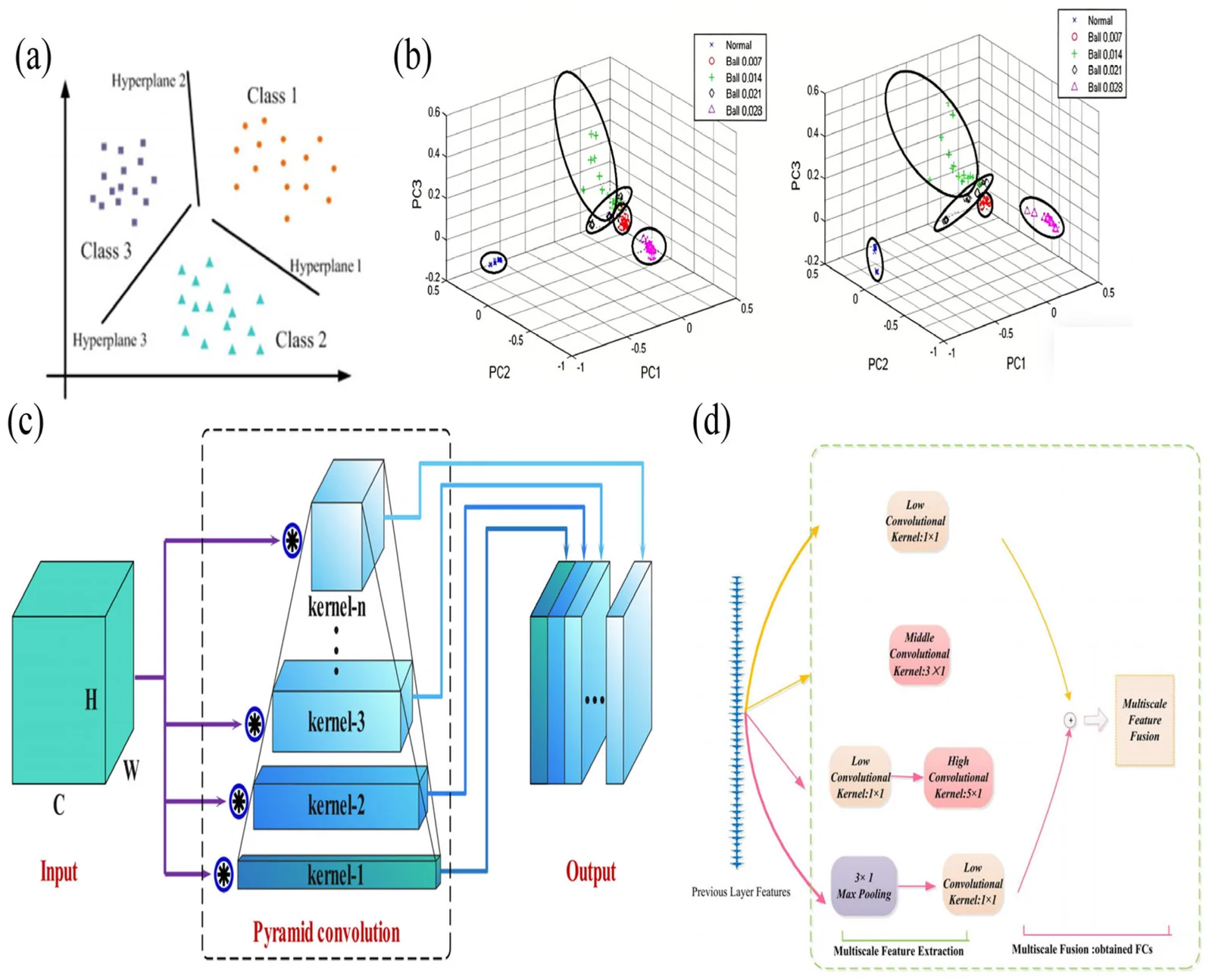
Traditional machine-learning-based bearing fault diagnosis. (**a**) Multi-classification method, reproduced with permission from [72], copyright 2019, Springer; (**b**) Scatter plots of data used in rolling element fault level identification case, reproduced with permission from [73], copyright 2014, Springer; (**c**) Structure of the bidirectional spatial pyramid convolution module, reproduced with permission from [81], copyright 2025, Springer; (**d**) Improved multi-scale fusion feature module, reproduced from [82] under CC BY 4.0.

For instance, as illustrated in Figure 5a,b, Jiang et al. and Ziani et al. introduced feature selection algorithms to filter fault-sensitive features prior to inputting them into the SVM [72,73], thereby mitigating the impact of the curse of dimensionality. In the direction of multidomain feature fusion, researchers have combined time-frequency feature extraction with multidomain information fusion to construct more robust classification frameworks [74,75,76]. Although these optimizations have improved SVM accuracy to a certain extent, they essentially remain a fragmented architecture of manual feature engineering plus shallow models. Once the feature extraction stage fails, the classifier is incapable of self-correction, rendering SVMs inadequate for adapting to nonstationary signals under complex, variable operating conditions.

Furthermore, classic supervised learning algorithms such as the Relevance Vector Machine (RVM) [77], Extreme Gradient Boosting (XGBoost) [78], K-Nearest Neighbor (KNN) [79], and Random Forest (RF) [80] have also been extensively adopted in the field of bearing fault diagnosis. Together with SVMs, these algorithms constituted the core supervised learning methodology system for bearing fault diagnosis during the era of traditional machine learning, delivering excellent diagnostic performance under controlled operating conditions. However, when deployed in complex industrial settings, these methods encounter a fundamental bottleneck: a severe scarcity of labeled data. In industrial production environments, the failure of critical equipment components often leads directly to production line shutdowns and may even trigger major safety incidents. Consequently, acquiring a sufficient number of fault samples is physically almost impossible, and the exorbitant cost of data annotation further obstructs the pathway to data accumulation. Confronted with this dilemma, the academic community has gradually abandoned learning paradigms that rely entirely on labeled data, shifting instead toward exploring unsupervised learning paradigms. These methods do not depend on label information; instead, they automatically mine differences in data distribution directly from raw vibration or acoustic signals to achieve fault discrimination through clustering. This conceptual shift from relying on manual annotation to exploiting the intrinsic structure of data perfectly aligns with the practical reality of scarce labels in industrial settings.

The traditional Shallow Neural Network (NN) is a category of machine-learning models inspired by the information transmission mechanisms of biological neurons, typically comprising only one or two hidden layers. Incapable of autonomously mapping raw signals to high-level semantic features, these networks require the manual pre-extraction of handcrafted features such as time-domain and frequency-domain parameters as input. Fundamentally constituting a semi-automated scheme that combines feature engineering with a classifier, this architectural constraint limits their generalization capability when processing non-stationary and non-linear signals.

Unsupervised clustering addresses the scarcity of labeled data by automatically identifying fault patterns from the intrinsic distribution of unlabeled data. However, partitional clustering such as K-means is sensitive to initial centroid selection, requires a predetermined number of clusters, and is prone to local optima on irregular fault data. To address this deficiency, research initially focused on enhancing the robustness of K-means. Pan et al. [83] introduced a complex network node degree algorithm to optimize the initial centroid selection, characterizing sample similarity through topological structures, which significantly mitigated the risk of premature convergence. Zhang et al. [84] simplified feature inputs to merely three time-domain indicators, including energy and root mean square. They reconstructed the initial centroid strategy based on global mean and maximum Euclidean distance. Validated on the CWRU dataset, this method improved accuracy by 31.875% without misclassification between rolling element faults and normal samples, demonstrating its practical utility for rapid on-site industrial diagnosis. Although optimized, the physical limitation of K-means regarding preset cluster numbers cannot be eradicated. To this end, Zhu et al. adopted the Density-Based Spatial Clustering of Applications with Noise (DBSCAN) [85], utilizing its density-connectivity property to automatically identify fault clusters of arbitrary shapes and flag noise points, eliminating the need to predefine cluster counts. In simulations of low-speed wind turbine conditions (30 rpm), DBSCAN successfully overcame the challenge of distinguishing faults from raw waveforms under low-speed conditions, outperforming K-means significantly in clustering efficacy. Nevertheless, while unsupervised clustering eliminates dependency on labels, it encounters a new challenge: insufficient feature discriminability. Once manually extracted features fail to effectively represent the physical information of faults, clustering performance deteriorates substantially.

Regarding high-dimensional feature redundancy in bearing diagnosis, traditional linear dimensionality reduction methods such as Principal Component Analysis (PCA) can effectively reduce computational overhead by retaining core variance information [86,87]. However, the inherently linear projection mechanism of PCA prevents it from capturing complex nonlinear characteristics embedded within fault signals, leading to the loss of deep-layer fault information during the dimensionality reduction process. To overcome this physical limitation, the Autoencoder has emerged owing to its powerful nonlinear mapping capabilities. Through its encoding and decoding reconstruction mechanism, it successfully extracts deep features that possess significantly higher representational capacity compared to those derived from PCA [88]. This transition from linear dimensionality reduction to nonlinear feature learning further substantiates the distinct performance superiority of nonlinear unsupervised methodologies.

### 4.2. Deep-Learning-Based Fault Diagnosis Techniques

Traditional machine-learning methods are constrained by the inherent bottlenecks of manual feature engineering and shallow nonlinear mapping, resulting in insufficient generalization capability when processing increasingly complex non-stationary signals in industrial scenarios. This deadlock was definitively broken in 2006 when Hinton and Salakhutdinov [17] proposed a layer-wise pretraining framework, which successfully resolved the training difficulties of deep neural networks and propelled the field from theoretical conception into a stage of large-scale application. The core of this technological leap lies in the model’s ability to automatically learn multi-level feature representations directly from raw data. This fundamentally transformed the traditional machine-learning paradigm that relied on manual feature engineering, realizing a fundamental shift from experience-driven to data-driven approaches, and it directly catalyzed the subsequent explosive growth of deep-learning-based fault diagnosis methods.

**Figure 6:**
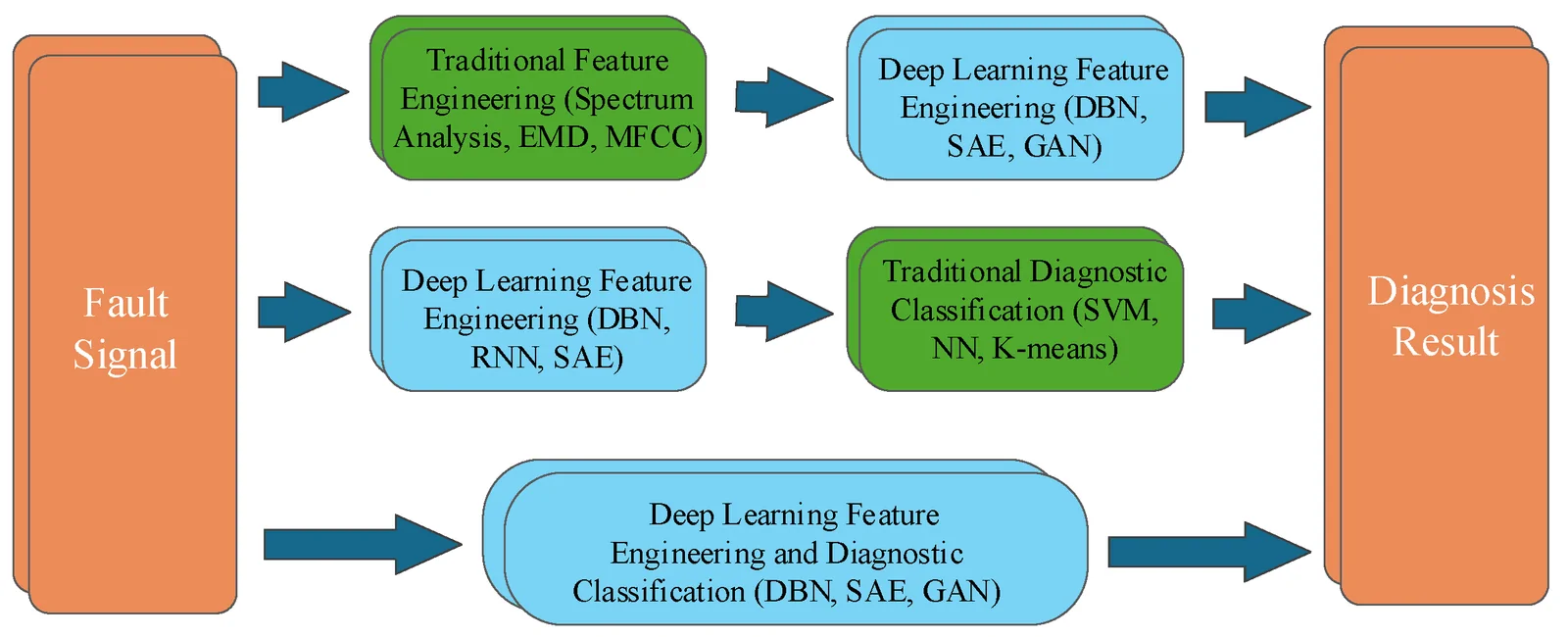
Comparison between traditional shallow machine-learning and deep-learning approaches.

Compared with traditional shallow machine-learning approaches, deep-learning methods demonstrate markedly superior technical advantages. First, they have dismantled the conventional paradigm that separates feature extraction from intelligent diagnosis. Without relying on sophisticated signal preprocessing techniques or extensive domain-specific expertise, these methods realize an end-to-end integration of the fault diagnosis workflow. Second, empowered by deep network architectures comprising multiple non-linear mapping layers and stacked hidden layers, these approaches can automatically excavate abstract features with potent fault representation capabilities directly from raw data. This enables highly efficient adaptation to the inherently non-stationary and non-linear characteristics of mechanical vibration signals, as exemplified by the typical deep-learning-based fault diagnosis scheme illustrated in Figure 6. Currently, the Autoencoder [89], Deep Belief Network (DBN) [90], Convolutional Neural Network [18], and Recurrent Neural Network (RNN) [91] constitute the core network architectures of classical deep learning. In recent years, these architectures have been successfully deployed by numerous researchers in the field of bearing fault diagnosis.

To organize the extensive literature, three orthogonal dimensions are distinguished: (i) the learning paradigm, namely supervised, unsupervised, and semi-supervised learning, which concerns the amount and form of label supervision; (ii) the model architecture, such as RNN, CNN, autoencoder, GAN, GNN, and Transformer, which can in principle be trained under any paradigm; and (iii) the knowledge transfer strategy, such as transfer learning, which is largely independent of both the architecture and the paradigm. The discussion below follows the paradigm dimension for readability while explicitly noting the architecture and transfer dimensions, so that methods belonging to multiple categories are not misassigned.

**Figure 7:**
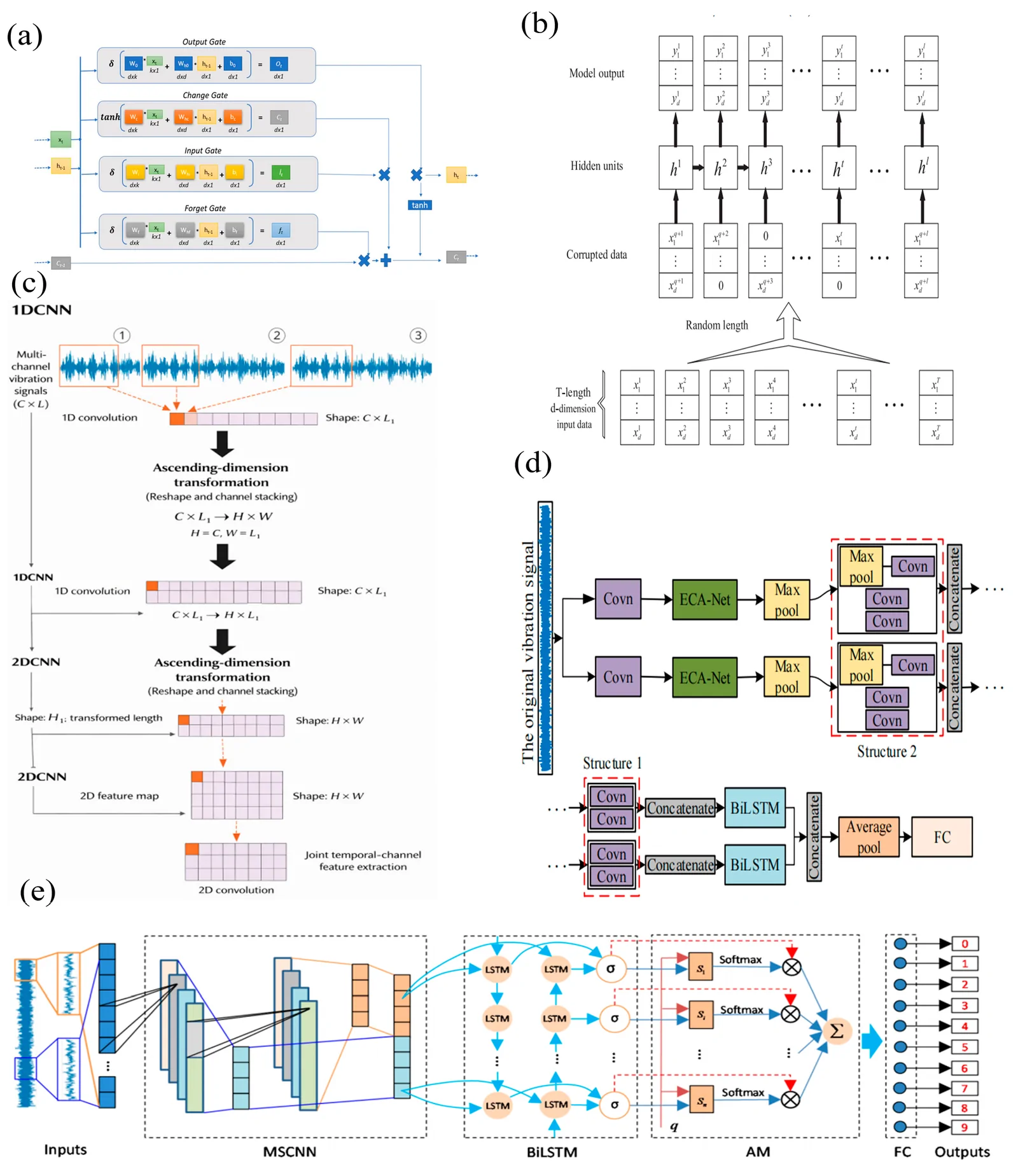
Representative deep-learning architectures for bearing fault diagnosis. (**a**) LSTM cell structure, reproduced with permission from [92], copyright 2023, Springer; (**b**) Schematic diagram of GRU-NP-DAE modeler, reproduced with permission from [93], copyright 2018, Elsevier; (**c**) Schematic diagram of channel dimension promotion, reproduced from [96] under CC BY 4.0; (**d**) Structure diagram of 1DMCICNN, reproduced from [97] under CC BY 4.0; (**e**) MSCNN-BiLSTM-AM Network Structure, reproduced from [100] under CC BY 4.0.

The Recurrent Neural Network (RNN), dating back to the Elman network of 1990 [19] has been applied to rolling bearing diagnosis mainly for capturing temporal degradation patterns and suppressing noise. For instance, Akcan et al. [92] employed the One-Dimensional Ternary Pattern (1D-TP) to extract UPPER and LOWER features, which were then input into an LSTM network. This approach bypasses complex time-frequency transformations to achieve accurate modeling of the degradation trajectory, as illustrated in Figure 7a. Confronting the challenges posed by intense industrial noise and multi-sensor fusion, Liu et al. [93] developed a Gated Recurrent Unit-based Nonlinear Predictive Denoising Autoencoder (GRU-NP-DAE). Utilizing gating mechanisms to process multi-source long sequences and implementing a variable-length input strategy, this model demonstrated superior diagnostic accuracy under strong noise and variable load conditions compared to traditional SVMs and shallow autoencoders, as shown in Figure 7b. To further exploit model potential, the academic community has transcended the limitations of single architectures by constructing cascaded hybrid models. For instance, Song et al. [94] proposed an LSTM-GRU-LSTM cascaded diagnostic model, leveraging CNNs for spatial feature extraction and RNNs for temporal dependency capture, thereby achieving a dual enhancement in accuracy and generalization. Subsequently, Song et al. [95] introduced an end-to-end framework featuring a cascade of fused modules combining a One-Dimensional Convolutional Neural Network (1D-CNN) with LSTM-GRU units. Its LSTM and GRU variants mitigate gradient vanishing through gating mechanisms; however, their inherently sequential computation limits efficiency and long-range memory capacity, which hinders real-time deployment on industrial big data.

Since the adoption of LeNet-5 in bearing diagnosis in 2016, CNNs have replaced manual feature engineering through hierarchical convolution-pooling cascades and become the mainstream model for adaptive feature extraction. However, standard CNNs suffer from static receptive fields, phase-erasing pooling, and fixed-length inputs, which limit their robustness under variable speed and strong noise; numerous enhancements have been proposed. As illustrated in Figure 7c, Bai et al. [96] proposed an Augmented Dilated Convolutional Neural Network (ADCNN), which enhanced noise immunity through large convolutional kernels while implementing model lightweighting strategies such as grouped convolution. Guo et al. [97] introduced a one-dimensional multi-channel improved CNN (1DMCICNN) methodology, as depicted in Figure 7d, integrating multi-scale convolution, Efficient Channel Attention (ECA) mechanisms, and Bidirectional Long Short-Term Memory (BiLSTM) networks to refine the traditional CNN model. Furthermore, Zou et al. [98] proposed a CNN model fusing gating mechanisms with multi-scale features, achieving synergistic optimization through multi-scale modules, dynamic gated convolution, and Squeeze-and-Excitation (SE) attention mechanisms. Ning et al. [99] proposed a Multi-Scale Dilated Attention CNN (MSDA-CNN), capturing cross-scale degradation features via multi-scale dilated causal convolutions and combining attention mechanisms to suppress noise interference. These methods consistently maintain high recognition accuracy under intense noise conditions. Regarding optimization for small samples and cross-domain generalization, researchers have opted to integrate CNNs with other deep-learning models. As shown in Figure 7e, Dengfeng et al. [100] proposed the Multi-Scale CNN (MSCNN) (Bidirectional Long Short-Term Memory (BiLSTM)) Attention Mechanism (AM) fusion model, incorporating transfer learning to achieve adaptive diagnosis in small-sample scenarios. Addressing the deficiency in temporal modeling capability of CNNs, Chen et al. [101] chose to integrate the adaptive feature extraction strengths of CNNs with the temporal modeling advantages of LSTMs, enabling end-to-end construction of bearing health indicators. The performance under variable operating conditions significantly surpassed that of standalone CNN or RNN models. Additionally, Kiakojouri et al. [102] utilized a generalized 1D-CNN model pre-trained on simulated data, completing model training using data from multiple bearing types to achieve efficient fault diagnosis across models, operating conditions, and scenarios. Their supervised nature also implies heavy dependence on large labeled datasets, which motivates the unsupervised and semi-supervised alternatives discussed below.

Although supervised learning algorithms, particularly those represented by Convolutional Neural Networks, have demonstrated remarkable classification performance in bearing fault diagnosis, real-world industrial scenarios present a significant challenge: equipment operates predominantly under normal conditions, resulting in a scarcity of fault samples, most of which remain unlabeled. Manual annotation is not only prohibitively expensive but also heavily reliant on domain-specific expert experience, rendering it impractical for meeting large-scale engineering demands. In contrast, unsupervised learning algorithms can autonomously excavate potential data structures and intrinsic correlations from unlabeled data, effectively mitigating the core challenge of scarce annotated data in industrial settings. This capability underscores their substantial application value in the field of bearing fault diagnosis. Representative methodologies in this domain extend beyond the previously discussed Principal Component Analysis (PCA) and Autoencoders to include Stacked Autoencoders (SAE) and Generative Adversarial Networks (GANs).

**Figure 8:**
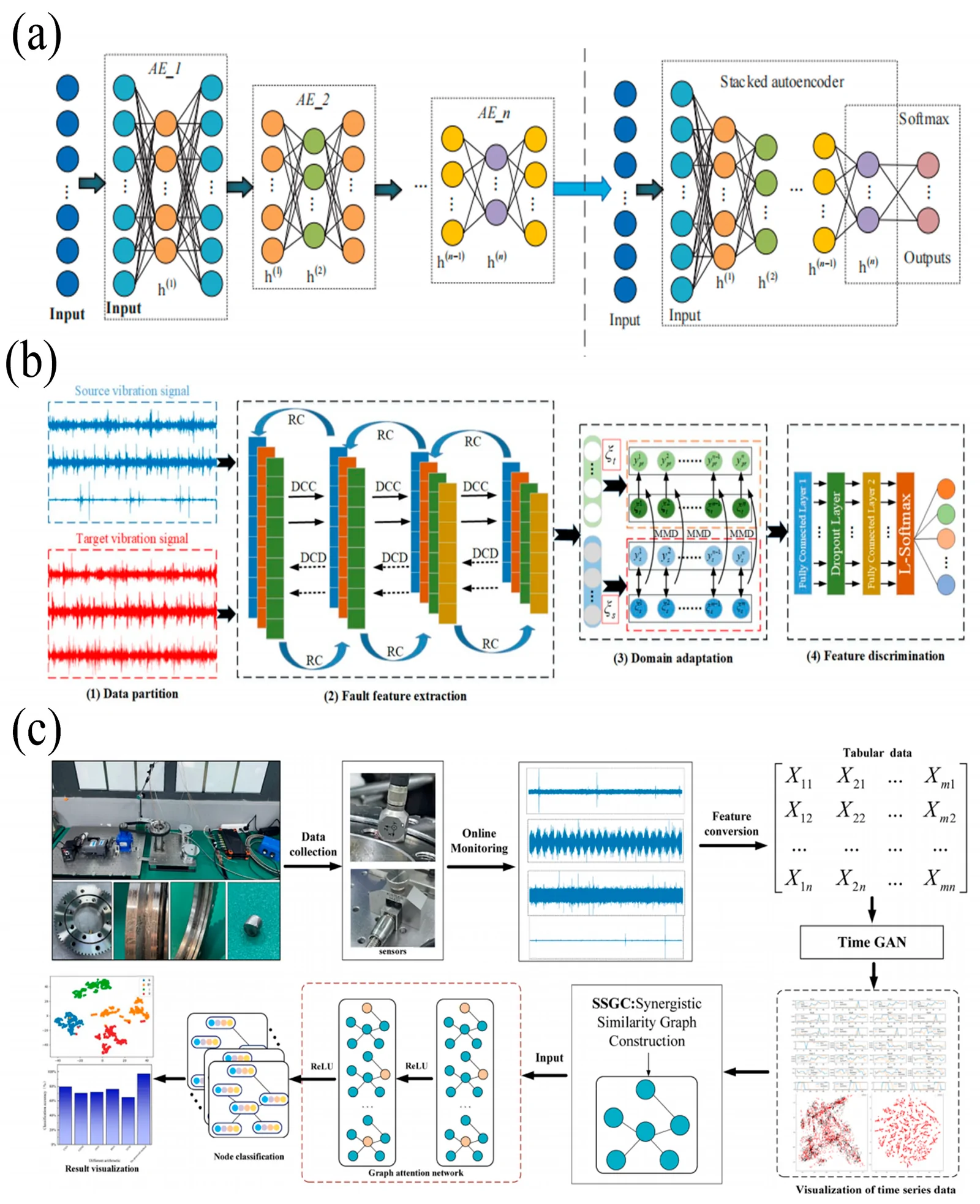
Stacked autoencoder-based unsupervised fault diagnosis. (**a**) Unsupervised training process of stack autoencoder and Supervised fine-tuning process of stack autoencoder, reproduced with permission from [103], copyright 2020, IEEE; (**b**) The framework structure of the TRDAE-TL model, reproduced with permission from [105], copyright 2025, Springer; (**c**) Framework of the proposed fault diagnosis methodology, reproduced with permission from [107], copyright 2025, Springer.

The Stacked Autoencoder (SAE) hierarchically stacks multiple autoencoders so that each layer encodes the output of the previous one, extracting high-order fault features in an unsupervised manner. Optimizations addressing its limited feature discriminability and temporal adaptability have been widely proposed. For instance, Cui et al. [103] proposed a serial architecture combining a Feature Distance Stacked Autoencoder (FD-SAE) with a Support Vector Machine (SVM). By introducing a feature-distance penalty term, this method enhanced the distinctiveness of fault features, effectively mitigating the issue of confusable rolling element faults, as illustrated in Figure 8a. In the pursuit of enhanced noise robustness, Xu et al. [104] opted to embed Lowess filtering layer-by-layer within the basic SAE framework to achieve denoising. This approach significantly reduced interference under strong noise conditions while enabling end-to-end feature extraction in label-free scenarios. Regarding temporal modeling and cross-domain adaptation, researchers have integrated SAEs with data-driven intelligent algorithms. As depicted in Figure 8b, Shang et al. [105] combined a Temporal Residual Denoising Autoencoder (TRDAE) with transfer learning, merging dilated causal convolutions and residual structures to augment temporal feature extraction capabilities.

The Generative Adversarial Network (GAN), proposed in 2014 [106], learns the distribution of fault data through adversarial training and synthesizes high-fidelity samples, effectively addressing fault-sample scarcity and dataset imbalance, and it has been extensively adopted in the field of bearing fault diagnosis since 2017. For instance, as illustrated in Figure 8c, Sun et al. [107] integrated a Temporal Generative Adversarial Network (TimeGAN) with a Graph Attention Network (GAT) to generate high-fidelity temporal fault samples, thereby expanding the dataset and achieving high-precision diagnosis across multiple operating conditions in few-shot scenarios. Huang et al. [108] proposed an Improved Cycle-Consistent Generative Adversarial Network (ICycleGAN), utilizing spectral normalization to stabilize training and generate high-quality time-frequency image samples to balance the dataset. In the direction of optimizing sample quality and model performance, Luo et al. [109] introduced an Enhanced Relative Generative Adversarial Network (ERGAN). By employing a relative loss function with gradient penalty and spectral normalization to refine the training process, this method generates high-quality fault samples while effectively enhancing diagnostic robustness. Rathore et al. [110] proposed a Stacked Autoencoder-guided Walsh-GAN (SAE-WGAN), which incorporates the Wasserstein distance to stabilize training; the quality of samples generated by this model significantly outperforms those from traditional GANs and Variational Auto-Encoders (VAEs). Furthermore, in the expanded application of bearing reliability assessment, Meng et al. [111] proposed a reliability evaluation method combining GAN-based sample augmentation with the maximum entropy principle. By utilizing GANs to generate full-life-cycle vibration samples to expand the dataset, this approach achieved the precise segmentation of bearing life stages and accurate prediction of Remaining Useful Life (RUL).

However, the original GAN framework is constrained by inherent limitations, including a propensity for mode collapse, gradient vanishing, and pronounced training instability. Furthermore, its complex network architecture and the high difficulty associated with hyperparameter tuning pose significant practical obstacles to its deployment.

Its original framework nevertheless suffers from mode collapse, gradient vanishing, and training instability, which hinder practical deployment. For instance, Zhang et al. [112] proposed a diagnostic method based on an improved Convolutional Restricted Boltzmann Machine (CRBM). By optimizing feature reconstruction accuracy through zero-padding and integrating maximum likelihood estimation with cross-entropy loss, this approach effectively enhances fault feature extraction capabilities. Similarly, Zheng et al. [113] integrated Ensemble Empirical Mode Decomposition (EEMD) with DBN. In this framework, EEMD is utilized to decompose and reconstruct the signal, constructing an acceleration-velocity feature matrix as input for the DBN. This methodology enables the synchronous identification of both bearing fault types and the extent of damage.

To address the limitations of insufficient classification accuracy in unsupervised learning, semi-supervised learning integrates the dual advantages of supervised and unsupervised paradigms. This approach is highly compatible with typical industrial scenarios characterized by an abundance of unlabeled data and a scarcity of annotated fault samples. It effectively resolves the dual bottlenecks of high annotation costs in purely supervised learning and inadequate precision in purely unsupervised learning, positioning it as a central research hotspot in the field of intelligent bearing fault diagnosis. Among the architectures frequently adopted in this paradigm, Graph Neural Networks (GNNs) and Transformers have shown particularly strong results in semi-supervised settings; it should be emphasized, however, that these are architectures rather than semi-supervised methods, and both can equally be trained under supervised, self-supervised, or unsupervised settings. A cross-cutting strategy orthogonal to both architecture and paradigm, transfer learning, is discussed at the end of this section.

**Figure 9:**
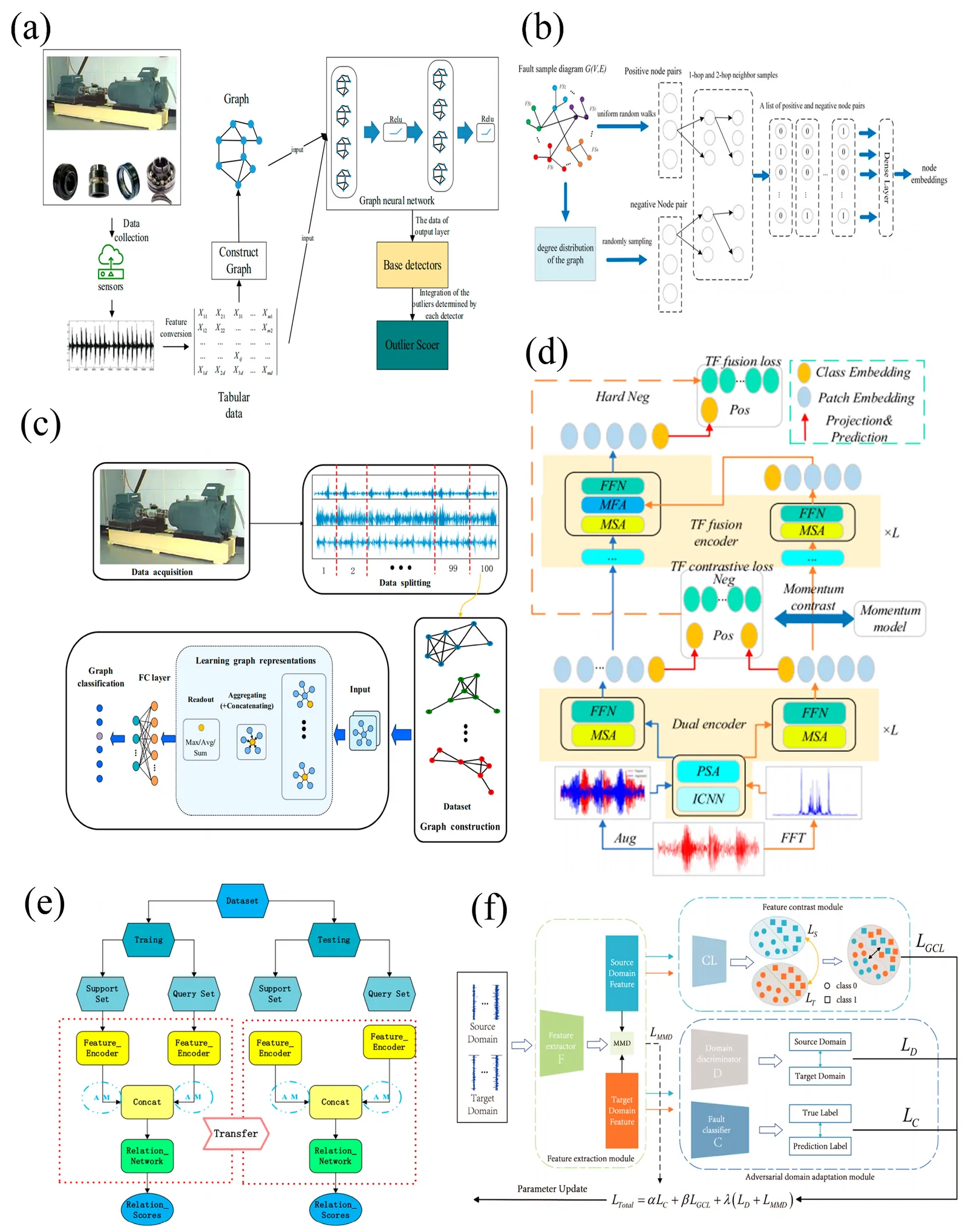
Graph neural network-based semi-supervised bearing fault diagnosis. (**a**) Flow chart of bearing fault detection based on graph neural network, reproduced from [116] under CC BY 4.0; (**b**) Learning node embedding by Graph Sample and Aggregate in an unsupervised way, reproduced with permission from [117], copyright 2025, Springer; (**c**) The framework of the proposed method, reproduced with permission from [21], copyright 2023, Springer; (**d**) Brief workflow of TFDDCF, reproduced with permission from [61], copyright 2025, Springer; (**e**) The structure of the MTLRN-AM, reproduced with permission from [122], copyright 2023, Springer; (**f**) Structure of CDAN, reproduced with permission from [124], copyright 2026, Springer.

The Graph Neural Network (GNN) is a deep-learning model specifically designed for processing graph-structured data. Its core principle involves aggregating neighborhood information and updating node features to learn the correlational characteristics between individual nodes and the global graph structure. This architecture is inherently suited for modeling non-Euclidean data. In semi-supervised scenarios in particular, GNNs can accomplish full-graph classification using only a minimal set of labeled nodes, which makes them well suited to industrial conditions characterized by scarce annotations; the same architectures are equally applicable under fully supervised settings. For instance, Gao et al. [114] and Yang et al. [115] successively proposed diagnostic methods integrating GNN with one-shot learning. In these approaches, Convolutional Neural Networks first extract features, after which the GNN constructs feature-association relationships and performs distance metrics and classification within non-Euclidean spaces, enabling efficient diagnosis under variable operating conditions with small samples. Addressing the difficulty of identifying early-stage incipient faults, Xiao et al. [116] adopted a GNN-based fault detection method (GNNBFD), as illustrated in Figure 9a. This method constructs similarity graphs using time-frequency features and integrates node and neighborhood information to achieve ensemble detection, maintaining a detection rate above 95% across multiple fault types. To tackle the challenge of scarce labeled data, Feng et al. [117] implemented an unsupervised Graph Sample and Aggregate-based fault diagnosis method, as shown in Figure 9b. A fault graph is constructed via K-nearest neighbor relations, and topological relationship features are extracted in an unsupervised manner before being fed to a classifier for final fault identification. To remedy the neglect of intrinsic data correlations in conventional methods, Ning et al. [21] proposed a GNN diagnostic method that converts time-series signals into graph structures, as shown in Figure 9c, achieving end-to-end high-precision diagnosis without requiring complex preprocessing. Furthermore, Liu et al. [118] implemented a bearing fault diagnosis method combining a simplified Graph Convolutional Neural Network with semi-supervised meta-learning. Treating each spectral sample as a graph node and utilizing K-Nearest Neighbors (KNN) to construct node-adjacency relationships, this approach transforms the bearing fault classification task into a graph node classification problem. The accuracy under variable operating conditions showed improvement compared to four mainstream algorithms.

The Transformer was proposed in 2017 [22]. Abandoning the sequential structure of traditional Recurrent Neural Networks (RNNs), it has become a central paradigm for temporal modeling owing to its parallelized computation capability and proficiency in capturing global contextual dependencies. Its self-supervised pretraining regime, followed by fine-tuning with limited labels, provides a natural route to semi-supervised deployment in industrial scenarios characterized by scarce labeling. For instance, He et al. [61] proposed an ICNN-Transformer dual-encoder self-supervised method. As illustrated in Figure 9d, this approach extracts global key features via peak sparse attention and accomplishes pretraining using contrastive loss, achieving the highest diagnostic accuracy of 99.57% under scenarios with limited labeled data. Addressing the inherent defect that the vanilla Transformer struggles to directly process one-dimensional vibration signals, Zhao et al. [119] proposed the Frequency Domain (FD) Transformer model. This architecture preprocesses signals via multi-channel one-dimensional CNNs before extracting features through densely connected Transformer encoders. Furthermore, focusing on time-frequency domain feature fusion and noise-robustness optimization, Qiu et al. [120] and Dong et al. [121] designed a bidirectional input mechanism and a time-frequency dual-channel dynamic fusion Transformer method, respectively. These innovations enhanced diagnostic accuracy under intense noise conditions while concurrently maintaining real-time inference efficiency.

The Transformer architecture demonstrates pronounced application advantages in the field of industrial fault diagnosis, primarily attributable to its exceptional global feature extraction capability and robust generalization performance under time-varying operating conditions. However, it remains constrained by inherent limitations, specifically suboptimal computational efficiency and a restricted capacity for positional information representation. To address these limitations, lightweight Transformer variants tailored to bearing diagnosis have been developed; a quantitative comparison of their computational complexity is provided in Section 6.3.

Transfer learning is a training strategy that is largely orthogonal to both the model architecture and the learning paradigm: it can be applied to CNNs, Transformers, or GNNs alike, under supervised or semi-supervised settings. Transfer Learning (TL) transfers generic knowledge from source tasks to data-scarce target tasks, reducing training costs and improving generalization across operating conditions and equipment. For instance, in the domain of few-shot rapid adaptation, Wei et al. [122] proposed the Meta-Transfer Learning Residual Network (MTLRN)—Attention Mechanism meta-transfer learning method. As illustrated in Figure 9e, this approach integrates the pretraining capacity of transfer learning with the rapid adaptation capability of meta-learning, demonstrating superior generalization performance under small-sample and variable operating conditions compared to conventional models. Similarly, Zeng et al. [123] introduced the Domain-Regularized Transfer Learning (DRTL)—Novel Gated Recurrent Unit (NGRU) meta-transfer learning method. Regarding cross-domain adaptation, Pan et al. [124] developed the Contrastive Domain Adaptation Network (CDAN) framework, which integrates contrastive learning with domain adversarial training. As depicted in Figure 9f, this framework aligns the distributions of labeled source domains and unlabeled target domains through an adversarial domain adaptation module while simultaneously incorporating a feature contrast module. This effectively mitigates the issues of low diagnostic accuracy and weak generalization in cross-domain fault diagnosis under variable operating conditions, achieving average accuracies of 99.64% and 80.25% on the CWRU and PU datasets, respectively. Furthermore, Song et al. [125] proposed a lightweight CNN transfer approach, achieving cross-condition and cross-domain adaptive diagnosis through feature enhancement and distribution alignment strategies. Zhang et al. [126] introduced the Adaptive Channel Enhancement Sparse Optimization Method lightweight transfer learning method, which maintains diagnostic accuracy while reducing model complexity through joint strategies involving adaptive channel enhancement, weight pruning, and domain adaptation loss. Additionally, in the refinement of traditional machine-learning models, Chen et al. [127] proposed an improved Least Squares Support Vector Machine (LSSVM) transfer learning method, effectively resolving the poor generalization performance inherent in traditional machine-learning models.

However, Transfer Learning remains constrained by inherent limitations, including a pronounced susceptibility to negative transfer, considerable difficulties in adapting to large domain discrepancies, and limited model interpretability.

## 5. Acoustic Signal-Based Bearing Fault Diagnosis

Although vibration signals constitute the predominant data source for rolling bearing fault diagnosis and have been extensively investigated, they are inherently subject to several unavoidable core limitations. Fault-induced impulses must propagate through multiple mechanical interfaces before reaching the sensor, frequently resulting in the attenuation and distortion of high-frequency, weak characteristic features. Consequently, acquisition efficacy is highly dependent on sensor installation location and technique [128], elevating the risk of missed detection of critical features. Furthermore, the requirement for intrusive sensor installation imposes practical constraints. These shortcomings serve as the primary impetus for introducing alternative signal sources, such as acoustic and current signals, to facilitate collaborative diagnosis.

It is crucial to note that the term acoustic signal broadly encompasses two modalities with fundamentally distinct physical essences. Specifically, Acoustic Emission signals refer to elastic waves propagating within solids, necessitating contact-based sensors for acquisition. In contrast, airborne acoustic signals represent a non-contact measurement modality, characterized by air vibrations induced by fault impacts, typically within the frequency range of 20 Hz to 20 kHz. Given the essential disparities in their physical nature and measurement characteristics, the term acoustic signal in this work specifically refers to airborne acoustic signals. For clarity, Table 1 contrasts the three physically distinct measurement modalities relevant to this review. These disparities in propagation media and paths are also the physical root of the cross-modal alignment challenges analyzed in Section 6.1.

The fusion-related implications of the vibration and airborne paths are analyzed in Section 6.1.1.

### 5.1. Machine-Learning Approaches Based on Acoustic Signals

The evolution of acoustic signal-based fault diagnosis technology is essentially a process of leveraging the non-contact nature of sound wave radiation to compensate for the shortcomings of traditional vibration monitoring. Unlike vibration signals, which require contact-based sensors to capture mechanical shocks transmitted through solid structures, airborne acoustic signals can capture broadband sound waves excited by fault impacts without contacting rotating components. This approach avoids the installation constraints and the susceptibility to oil contamination and electromagnetic interference of contact sensing and, under favorable signal-to-noise conditions and sensor-to-source distances, may offer higher sensitivity to incipient and weak faults that are heavily attenuated along the structural transmission path, as reported by the studies surveyed below. Based on these physical characteristics, diagnostic technology has evolved from relying on manual expertise within machine-learning paradigms to adopting data-driven deep-learning frameworks, thereby establishing a unique implementation pathway. A small number of the surveyed studies concern gears or pumps rather than bearings. They are retained as methodological references because their sensor-modality pipelines, namely microphone array processing and end-to-end acoustic learning, are component-agnostic and transfer directly to bearing acoustics; they are marked as such wherever they appear.

**Figure 10:**
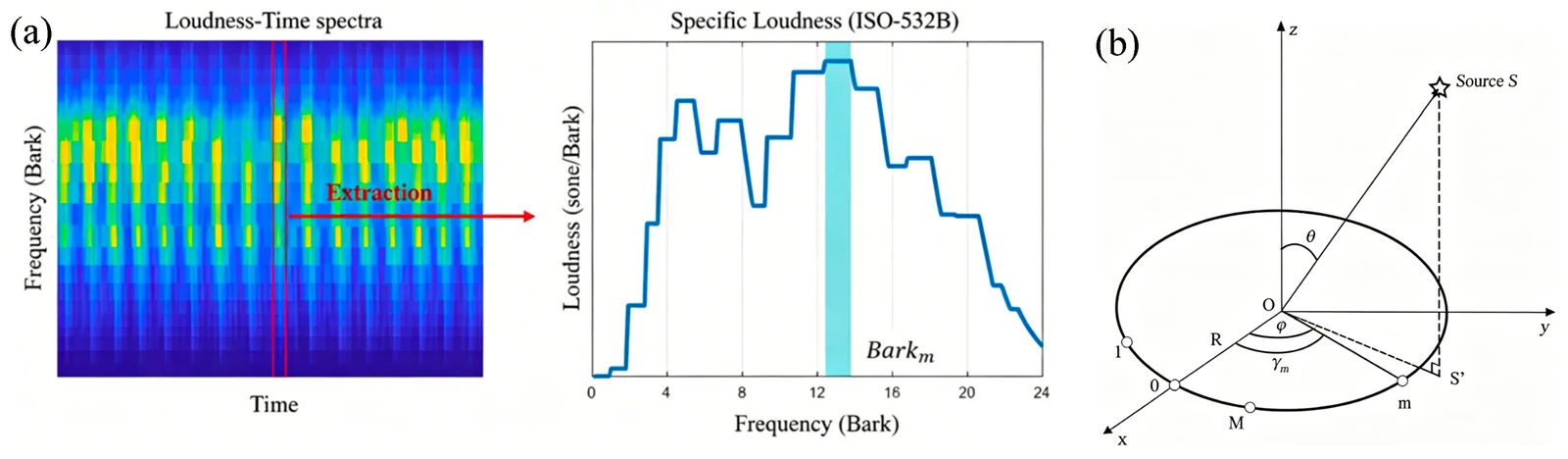
Acoustic feature extraction for bearing fault diagnosis. (**a**) Specific loudness and Barkm, reproduced with permission from [130], copyright 2026, Springer; (**b**) Far-field model with UCA for gear fault diagnosis, reproduced with permission from [133], copyright 2023, Springer.

In early methodologies based on traditional machine learning, the core logic involved utilizing signal processing algorithms to extract acoustic features from strong background noise, thereby replacing the precise analysis of fault characteristic frequencies required in vibration signals. Due to the susceptibility of acoustic signals to environmental reflection and multi-source aliasing interference, a primary focus of traditional technical routes has been the enhancement of the signal-to-noise ratio. Rather than relying solely on Fourier transforms, scholars have concentrated on adaptive algorithms such as Variational Mode Decomposition and Empirical Mode Decomposition, integrated with spectral kurtosis or loudness features for denoising, aiming to restore obscured fault impulse characteristics. The relative strengths of such adaptive decomposition methods on nonstationary and noise-corrupted signals have been systematically compared on a well-documented experimental benchmark by Civera and Surace [129]. For instance, Cong et al. [130] proposed a loudness-based adaptive filtering method (L-FPD), as illustrated in Figure 10a, which replaces conventional time-domain and frequency-domain analyses with loudness features to reduce noise and enhance fault impulse characteristics from the perspective of acoustic perception. Wu et al. [131] employed envelope spectrum analysis combined with Adaptive Variational Mode Decomposition (AVMD) denoising and Improved Multi-Verse Optimizer (IMVO)-optimized Maximum Correlated Kurtosis Deconvolution (MCKD) to stably extract composite fault features under strong noise conditions of −8 dB, achieving effective separation of inner and outer race composite faults in acoustic signals. Zhang et al. [132] conducted theoretical simulation research on bearing fault localization using an L-shaped microphone array based on the Two-Dimensional Multiple Signal Classification algorithm, systematically analyzing the influence of array element quantity, spacing, and signal-to-noise ratio on localization resolution. Furthermore, Li et al. [133] proposed a gear fault acoustic diagnosis method utilizing a Uniform Circular Microphone Array (UCA) combined with Ensemble Empirical Mode Decomposition and the Estimation of Signal Parameters via Rotational Invariance Techniques (ESPRIT), as shown in Figure 10b, which not only achieved fault diagnosis but also addressed the challenge of spatial fault localization, a capability that conventional vibration analysis attains only with dense sensor networks. It is noteworthy that although these methods eliminate the dependency on contact-based installation, the complex mapping relationship between acoustic features and fault mechanisms limits their generalization capability.

### 5.2. Deep-Learning Approaches Based on Acoustic Signals

With the advent of deep-learning methodologies, the diagnostic paradigm has shifted from manually engineered features to end-to-end automatic representation, significantly unlocking the potential of acoustic signal diagnosis. Unlike vibration-based approaches that primarily utilize Convolutional Neural Networks to extract local spatial features, acoustic signal diagnosis places greater emphasis on leveraging deep-learning models to capture the time-frequency evolution patterns and spatial propagation characteristics of sound waves. At the denoising level, deep-learning methods no longer rely on fixed filtering rules. Instead, they construct mappings between noisy and clean signals in a data-driven manner. For instance, Ding et al. [134] addressed the issue of airborne acoustic signals from wind turbine rotating bearings being contaminated by industrial background noise by proposing the WGU-Net deep-learning end-to-end denoising method. Through a modified U-Net architecture, this method automatically learns the mapping relationship between noisy and clean signals without requiring manual design of denoising rules or features. Similarly, Yong et al. [135] proposed an end-to-end stacked CNN method for gear fault diagnosis that directly inputs raw time-frequency acoustic signals, allowing the network to adaptively complete multi-channel information fusion and fault feature extraction without manual feature engineering, maintaining an accuracy rate above 96% under varying operating conditions. This effectively resolves the extreme low signal-to-noise ratio challenge, which is less common in vibration signals.

However, a systematic examination of the noise conditions adopted in these studies reveals a methodological concern: most denoising evaluations are conducted under a single or a narrow SNR setting, typically with stationary white or Gaussian noise added to clean recordings, which cannot represent the acoustic conditions in the field. As analyzed in Section 6.1.1, geometric spreading and atmospheric absorption rapidly weaken the direct sound with distance, while industrial scenes superimpose strong non-stationary noise sources; the effective SNR of a microphone placed 1 to 3 m from the bearing can therefore easily fall to 0 dB or below. Denoising superiority demonstrated only at a high, single SNR does not guarantee performance in this low-SNR regime. Table 2 summarizes the noise conditions used in representative acoustic denoising studies, from which it can be seen that multi-level low-SNR evaluation remains the exception rather than the rule. We therefore propose a standardized multi-SNR evaluation protocol for acoustic denoising algorithms: (i) construct at least four SNR groups covering the low-SNR regime, e.g., 10, 5, 0, and −5 dB, supplemented by injection of field-recorded industrial noise rather than synthetic white noise alone; (ii) report both signal-level metrics, such as SNR improvement and waveform correlation, and diagnosis-oriented metrics, such as the prominence of the fault characteristic frequency in the envelope spectrum and the classification accuracy of the downstream diagnostic model after denoising; and (iii) evaluate at multiple source-microphone distances, since the SNR condition is distance-dependent according to the propagation model. This protocol directly reflects the degradation physics of acoustic signals and provides a fair basis for comparing denoising methods.

To complement the methodological comparison, representative benchmark case studies of acoustic diagnosis are summarized in Table 3 and Table 4, covering the methods, benchmark datasets, diagnostic tasks, and reported accuracies (Table 3), together with the experimental conditions under which they were obtained, including the monitored machines, fault types and severities, microphone configurations, sensor-source distances, sampling parameters, operating conditions, and environments (Table 4). It should be emphasized that accuracy values reported by different studies cannot be compared directly, because they differ in datasets, class definitions, noise levels, train/test protocols, operating conditions, and sensor configurations, as reflected in Table 2, Table 3 and Table 4. This is precisely why the standardized multi-SNR protocol proposed above and the recommendation of reporting RT60 and source-array distance (Section 5.3) are needed to make future cross-study comparisons meaningful.

Noise conditions and denoising approaches are provided in Table 2; diagnostic models, tasks, and reported accuracies in Table 3; and computational complexity of the representative models in Section 6.3.

The reported results nevertheless reveal consistent trends: deep-learning models achieve over 90% accuracy on laboratory acoustic benchmarks under moderate noise, few-shot LLM-based diagnosis maintains high accuracy with only a handful of labeled samples [23], and accuracy under strong noise or reverberant field conditions remains the key differentiator among methods.

**Figure 11:**
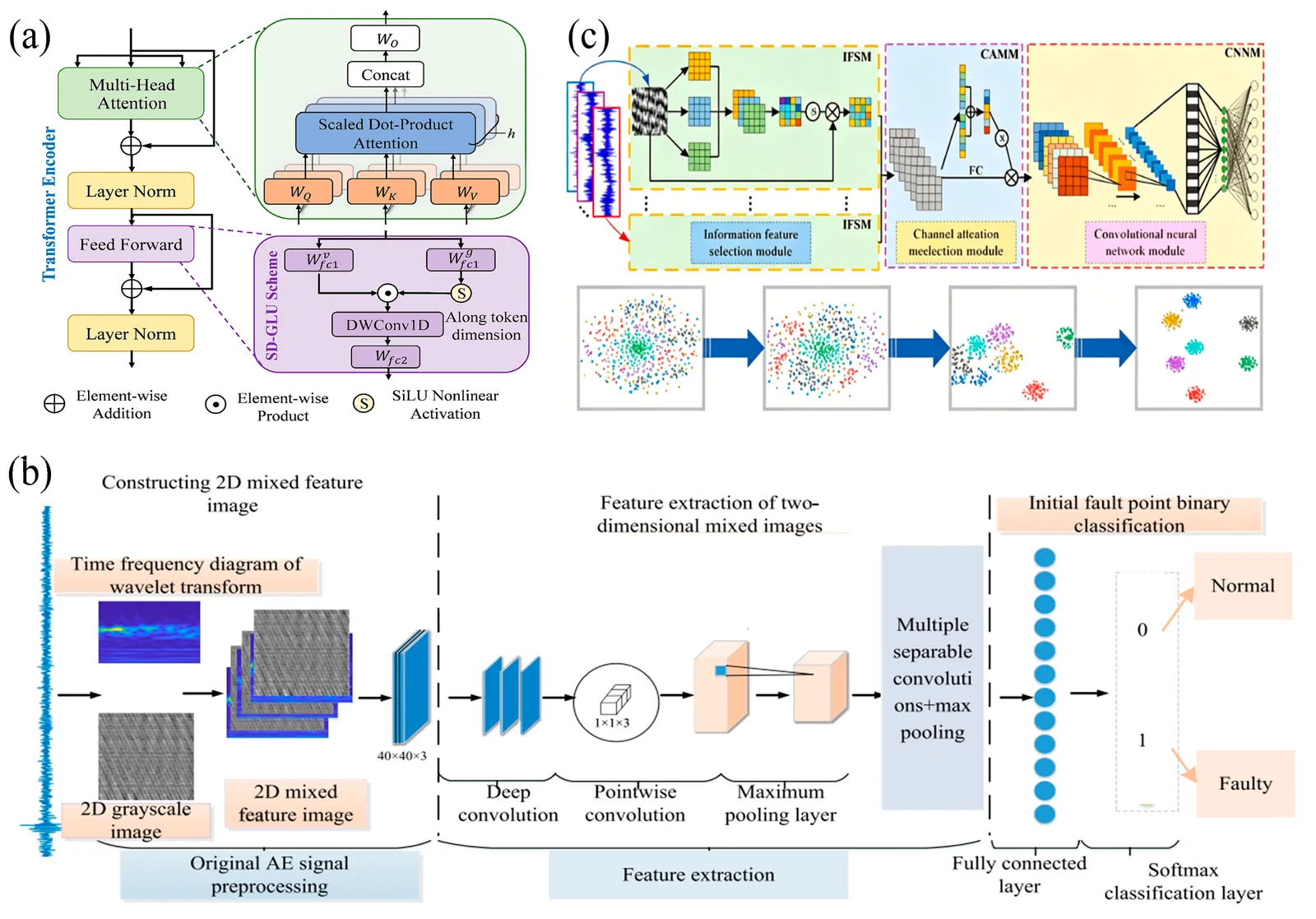
Transformer-based bearing fault diagnosis. (**a**) Detailed architecture of the Transformer encoder, reproduced with permission from [137], copyright 2026, Elsevier; (**b**) Structure chart of the 2D-GWCNN classifier for AE signals, reproduced with permission from [138], copyright 2026, Springer; (**c**) Enhanced Feature Extraction Network based bearing fault diagnosis, reproduced from [136] under CC BY 4.0.

At the diagnostic model level, as illustrated in Figure 11a, Peng et al. [137] proposed a lightweight ASFE (Acoustic Signal Feature Extraction) Transformer tailored to the characteristics of long acoustic sequences. By employing Analytic Amplitude-Phase Representation (AAPR) to preserve acoustic instantaneous dynamics, an Acoustic Frame Embedding Module (AFEM) to streamline sequences and reduce computational load, and an Split-Depth Gated Linear Unit module fused with deep convolution to enhance local features, the model maintained an accuracy of 96.85% even under extreme noise conditions of −5 dB. This approach leverages self-attention mechanisms to capture long-distance acoustic dynamic features, a capability unattainable by the short-term convolutional kernels used in traditional vibration analysis.

Furthermore, deep learning has effectively activated two complementary capabilities that are particularly relevant to acoustic signals. First, few-shot transfer capability: He et al. [23], on acoustic emission (AE) signals of full ceramic bearings, utilized the GPT-2 large language model, converting AE features into a text format to fine-tune the pre-trained GPT-2 model. This semantic understanding compensated for the scarcity of physically labeled acoustic data, enabling high-precision recognition of five fault types with only five labeled samples. Second, sensitivity to incipient faults: as shown in Figure 11b, Yu et al. [138] proposed the Adaptive Dual-Domain Quantile Discrepancy LSTM remaining useful life prediction method. This method converts acoustic signals into two-dimensional hybrid images, accurately identifies degradation points through Two-Dimensional Grouped Wavelet CNN and secondary discrimination, and completes prediction by combining adaptive dual-domain features with a reliability factor. This effectively addresses the difficulties in extracting weak incipient acoustic emission features and accurately locating initial degradation points. Finally, as depicted in Figure 11c, Koç et al. [136] further enhanced the recognition accuracy of composite faults by integrating multi-dimensional acoustic features through ensemble learning. However, it should be noted that the few-shot and cross-domain generalization results reported in most of these studies are benchmarked on standard laboratory datasets, predominantly the Case Western Reserve University (CWRU) bearing dataset, which contains only contact-measured vibration signals from a controlled test rig and no airborne acoustic channels. Whether such generalization gains persist under reverberant, multi-source industrial acoustic conditions remains an open question.

In contrast to vibration-based diagnosis, which exhibits a strong dependence on physical mechanisms and contact-based installation, machine-learning and deep-learning approaches utilizing acoustic signals demonstrate particular value, under favorable acoustic conditions, in non-contact monitoring, incipient fault warning, and array-based spatial localization; the latter is achieved by vibration sensing only through dense sensor networks, and its accuracy depends on the reverberation level and the array geometry discussed in Section 5.3. This is achieved primarily through the implementation of advanced noise-robust enhancement algorithms and the deep excavation of spatio-temporal features. However, acoustic signal diagnosis continues to face persistent challenges, notably the insufficient interpretability of “black-box” models and the distortion of features within highly reverberant environments. Addressing these limitations will be crucial for facilitating the successful industrial deployment of this technology in the future.

### 5.3. Reverberation Distortion and Calibration for Microphone Array Localization

Most microphone array localization methods reported in the literature, including beamforming, 2D-MUSIC [132], and circular array variants for bearings and gears [51,133], are developed and validated under idealized acoustic conditions, typically a free field or a low-reverberation laboratory in which only the direct propagation path is modeled. Industrial halls and equipment cabins, however, are highly reverberant: the reverberation time RT60 commonly reaches several seconds, and the critical distance, within which the direct sound dominates the reverberant field, shrinks to a few meters or less. Beyond this distance, the direct-to-reverberant ratio (DRR) falls below 0 dB, and three forms of signal distortion arise: (i) coherent late reflections arrive within the analysis window and bias the time-difference estimates on which localization relies; (ii) the covariance matrix estimated from reverberant recordings deviates from the free-field array manifold, causing subspace leakage and spurious peaks in MUSIC-type methods; and (iii) low-frequency room modes and early reflections smear the spatial spectrum and degrade angular resolution. These effects explain why localization accuracy reported in laboratory validation does not carry over directly to field deployment.

Corresponding calibration optimization schemes can be organized into four categories. (1) Array self-calibration: online estimation and compensation of microphone position, gain, and phase drift, which are inevitable after installation; blind calibration with known-geometry constraints or auxiliary calibration sources can maintain the array manifold close to its nominal form. (2) Acoustic environment calibration: online measurement of the room impulse response, RT60, and DRR, followed by matched-field processing with the measured transfer functions instead of the free-field manifold, and by dereverberation preprocessing, such as weighted prediction error filtering or spectral enhancement before localization. (3) Robust localization algorithms: SRP-PHAT, which is inherently robust to reverberation, direct-path dominance selection that identifies early arrivals, and Deep-Learning-based direction-of-arrival estimators trained on reverberant field data. (4) Physics-informed cross-modal calibration: the propagation model established in Section 6.1.1 provides a physically grounded reference because the vibration signal arrives through the solid path with negligible delay; cross-correlating the vibration impulse with the microphone channels can identify the direct acoustic arrival and separate it from reflections, thereby anchoring the time-difference estimation. We recommend that future localization studies report the RT60 and source-array distance of their test scenes together with localization accuracy, so that results obtained in different acoustic environments become comparable.

## 6. Future Perspectives

While current algorithms have validated the efficacy of acoustic signals for non-contact monitoring and early fault sensitivity, a multifaceted gap persists between laboratory algorithms and complex industrial scenarios, encompassing physical mechanism adaptation, model credibility, and engineering deployment. This section focuses on the core bottlenecks of existing technologies and outlines prospective breakthroughs across three dimensions: multi-modal deep fusion, trustworthy Large Language Model assistance, and engineering implementation. It aims to provide strategic insights for constructing a comprehensive, all-scenario bearing fault diagnosis system.

### 6.1. Multi-Modal Fusion Diagnosis

While acoustic signals alone are constrained by limitations such as acoustic wave occlusion, attenuation during long-distance propagation, and insufficient characterization of structural resonance-related faults, they can form complementary feature sets with vibration, current, temperature, and other signal modalities. Consequently, multi-modal collaborative fault diagnosis centered on acoustic signals has emerged as a pivotal pathway to transcend the performance ceiling of single-signal diagnosis and adapt to complex industrial scenarios. It represents a key advanced research direction in this field in recent years. The core objective of this approach is to achieve deep fusion of multi-source signals, leveraging the non-contact acquisition capability of acoustic signals and their reported sensitivity to incipient faults under adequate signal-to-noise conditions, alongside the operational condition characterization strengths of other signals. This strategy aims to mitigate diagnostic blind spots inherent in single-sensor approaches and realize a comprehensive, full-dimensional, and precise diagnosis of bearing faults.

#### 6.1.1. Physical Mechanism of Vibration–Acoustic Propagation and Feature Alignment

To design a physically meaningful fusion framework, it is essential to first clarify how a bearing fault signature manifests in the two modalities. A fault-induced impact acts as a common excitation source *s*(*t*) that simultaneously excites elastic waves within the solid structure and acoustic radiation into the surrounding air. The two observations can therefore be modeled as

(1) $$y_{v}\left(t\right)=h_{v}\left(t\right)*s\left(t\right)+n_{v}\left(t\right)$$

(2) $$y_{a}\left(t\right)=h_{a}\left(t\right)*s\left(t-\tau \right)+n_{a}\left(t\right),\;\tau =d_{m}/c_{a}$$

where $h_{v}(t)$ and $h_{a}\left(t\right)$ denote the structural and airborne transfer paths, respectively, $n_{v}(t)$ and $n_{a}\left(t\right)$ represent path-specific noise, $d_{m}$ is the source-microphone distance, and $c_{a}$ ≈ 343 m/s is the speed of sound in air. In contrast, elastic waves propagate in steel at approximately 5000 m/s, so the vibration-path delay is negligible, whereas the acoustic signal arrives at the microphone with a time lag *τ*, approximately 2.9 ms at $d_{m}$ = 1 m, corresponding to about 140 samples at a 48 kHz sampling rate. This delay is comparable to several impulse intervals of early faults and must be compensated before waveform-level fusion.

Equations (1) and (2) directly explain the core characteristics that govern cross-modal alignment. First, because the two paths share the same source, the envelope spectra of both signals contain the identical kinematic fault characteristic frequencies (BPFO, BPFI, BSF, FTF), e.g.,

(3) $$BPFO=\frac{N}{2}\cdot f_{r}\cdot \left(1-\frac{d}{D}cos\varphi \right)$$

For a fixed outer race, where N is the number of rolling elements, $f_{r}$ is the shaft rotation frequency, d is the ball diameter, D is the pitch diameter, and φ is the contact angle; these frequencies therefore provide natural anchors for cross-modal alignment. Second, the carrier bands differ: the vibration signal concentrates around structural resonance bands excited by the impacts (the physical basis of resonance demodulation techniques), whereas the airborne signal occupies a broadband acoustic radiation band (nominally 20 Hz–20 kHz). The two modalities thus share the same modulation frequency but carry it on different carriers, which is the physical foundation of their complementarity. Third, the path properties differ substantially: the solid path is dominated by multi-interface reflection and joint low-pass filtering, which attenuate and distort high-frequency impulse components, whereas the air path is governed by geometric spreading (sound pressure ∝ 1/r) and frequency-dependent atmospheric absorption (approximately proportional to f^2^), together with reflection and reverberation in confined spaces. Consequently, the reliability of each modality varies with distance, mounting, and environment; such information should be exploited as fusion confidence rather than treated as a nuisance. Table 5 summarizes these physical differences and their implications for fusion design.

**Figure 12:**
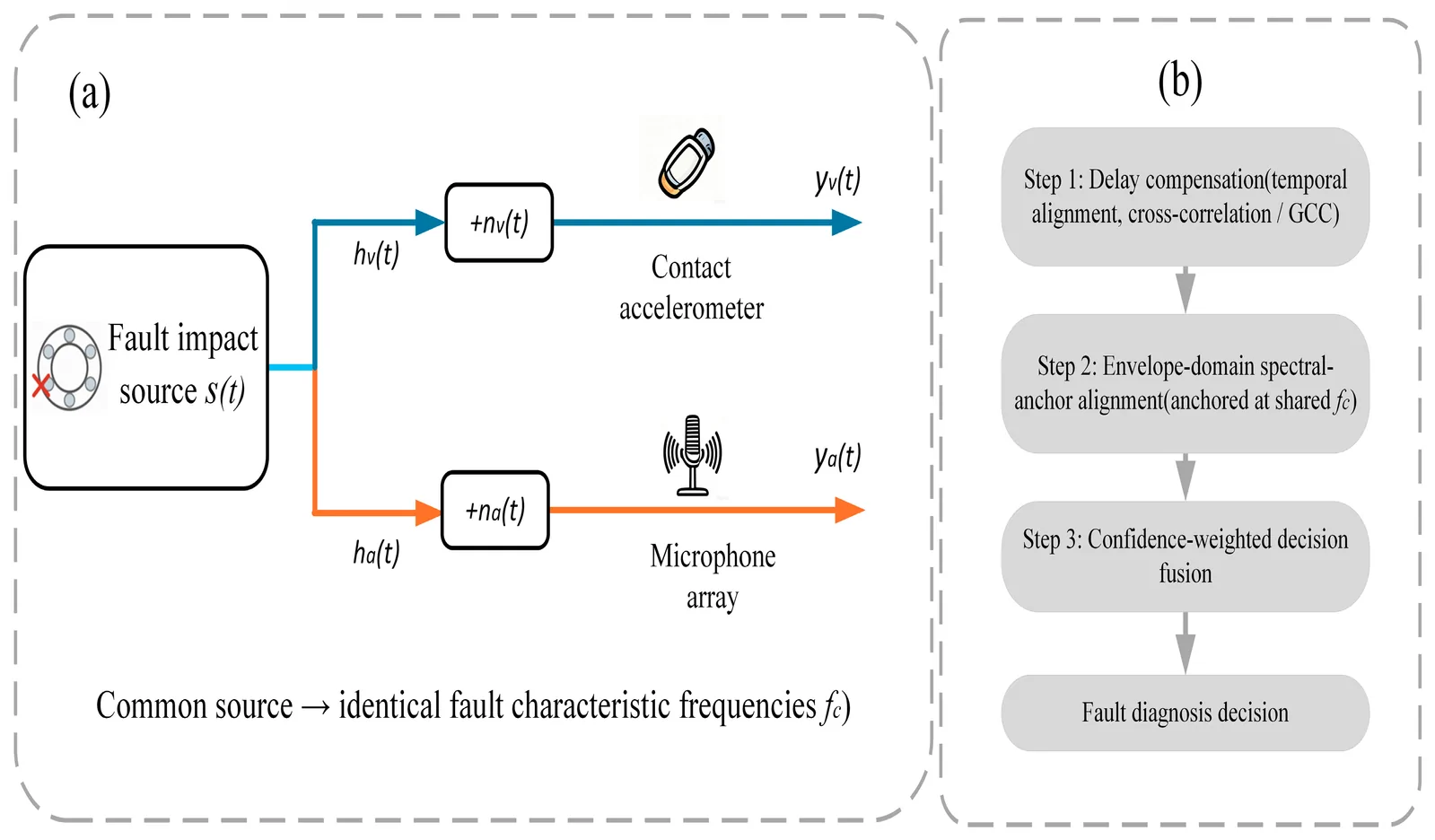
Physics-informed vibration–acoustic fusion framework: (**a**) common-source dual-path propagation model; (**b**) three-level feature alignment logic consisting of delay compensation, envelope-domain spectral-anchor alignment, and confidence-weighted decision fusion.

The above physical rules motivate a three-level feature alignment logic. (i) Temporal alignment: the propagation delay *τ* should be estimated, e.g., by cross-correlation or the generalized cross-correlation method and compensated before fusing the two streams; most existing vibro-acoustic fusion studies implicitly assume τ ≈ 0, which introduces misalignment between waveform segments. (ii) Spectral-anchor alignment: because the instantaneous phase of a signal is not preserved across heterogeneous propagation media, feature alignment should be performed in the envelope domain rather than the raw phase domain. The shared fault characteristic frequencies $f_{c}$ and their harmonics serve as alignment anchors: cross-modal attention mechanisms can align the envelope spectral peaks around $f_{c}$ while keeping the two carriers (structural resonance band for vibration, acoustic radiation band for airborne sound) as complementary channels. (iii) Confidence-weighted decision fusion: per-modality path attenuation and estimated SNR can be used to weight evidence at the decision level (e.g., in DSmT/D–S fusion), so that the modality with the cleaner propagation path dominates under a given operating condition. Figure 12 illustrates this physics-informed alignment framework, which is proposed here as a design blueprint for future fusion models.

#### 6.1.2. Existing Three-Level Fusion Approaches and Their Physical Limitations

Within the realm of multi-modal collaborative diagnosis, although the three-tier fusion framework comprising data-level, feature-level, and decision-level integration has been preliminarily validated, significant challenges remain unresolved. These include addressing the asynchrony in signal acquisition, achieving cross-modal feature alignment, and implementing redundant feature suppression. While data-level fusion provides a foundational multi-dimensional dataset, exemplified by the Cross-Attention Multi-modal Ensemble Time-Domain model proposed by Keshun et al. [139], which utilizes a compressed attention mechanism to dynamically perceive signal correlations and enhance model interpretability through adaptive weighted fusion or hybrid input strategies, achieving accuracies of 93.56% and 91.26% under 0 dB and −6 dB signal-to-noise ratios, respectively, the issue of dimensional mismatch and noise coupling continues to constrain the upper limit of fusion performance. Furthermore, Fang et al. [140] explored FFT-based cross-fusion methodologies within this layer. However, from the physical perspective established in Section 6.1.1, such data-level fusion schemes generally neglect the propagation delay τ between the two modalities, which can be regarded as blind fusion in the time domain.

Feature-level fusion, representing the mainstream research direction, has seen notable advancements. Wang et al. [141] utilized 1D-CNNs to independently extract features from vibration and acoustic signals, subsequently fusing them via fully connected layers to maintain high accuracy under low signal-to-noise ratios of −6 dB. Iqbal et al. [142] converted acoustic-vibration signals into time-frequency representations, employing parallel LeNet-5 CNNs for deep feature extraction, thereby further validating the efficacy of feature complementarity. Ding et al. [143] achieved a 95.42% accuracy rate in high-entropy, multi-noise environments through the Cross-Attention Vibro-Acoustic Fusion Network framework, which integrates Bidirectional Cross-Attention (BCA) with Causal Inference (CI). Nevertheless, these efforts necessitate deeper integration with the mechanical dynamics of vibration signals and the propagation attenuation laws of acoustic signals to construct deeply fused models endowed with physical interpretability. Nevertheless, directly concatenating dual-modal features without envelope-domain alignment anchored on the shared fault characteristic frequencies conflicts with the physical fact that instantaneous phase is no longer comparable across propagation media, which is identified here as a key reason for unstable fusion gains across operating conditions.

Regarding decision-level fusion, Jing et al. [144] realized three-tier adaptive fusion spanning data, feature, and decision levels via DCNNs. Chen et al. [145] elevated fusion accuracy to over 99% across four operating conditions by combining t-Distributed Stochastic Neighbor Embedding-ASC feature selection with the Dezert-Smarandache Theory. Concurrently, Xu et al. [146] attained a diagnostic accuracy of 97.94% on high-speed train axle box bearing datasets using a Dynamic Enhanced Weighted Voting Strategy (DEWVS). However, a critical frontier remains: the integration of fusion rules with real-time operational feedback to devise fault-tolerant decision-making protocols capable of handling partial multi-sensor failure. Resolving this is paramount for transitioning from mere availability to verifiable trustworthiness. The credibility of such evidence fusion would be further enhanced if evidence weights were conditioned on per-modality path attenuation and SNR estimates, as suggested by the propagation analysis in Section 6.1.1.

### 6.2. Large Language Model-Assisted Diagnosis

Beyond conventional end-to-end deep learning models, Large Language Models offer a novel pathway to address the interpretability deficit of traditional models through their knowledge reasoning capabilities. At present, most LLM-based diagnosis studies operate on vibration spectra or general industrial information rather than on airborne acoustic measurements, so this subsection is framed as a broader outlook for intelligent bearing diagnosis, with acoustic-specific evidence highlighted wherever available. It should also be stressed that interpretability and fluent post-hoc explanation are distinct: the former requires the reasoning to be traceable to signal evidence and physical knowledge and verifiable against expert ground truth, whereas the latter may produce plausible yet unfaithful text. However, their role must be clearly defined as a knowledge enhancement and decision support safety layer rather than a replacement for end-to-end real-time diagnostic systems. Current research has preliminarily validated feasibility: for instance, Wang et al. [147] pioneered the integration of the Qwen2-VL-7B multimodal LLM into industrial diagnostics, constructing the DiagLLM (Diagnostic Large Language Model) framework by fusing envelope spectrum images with expert knowledge, effectively mitigating the utility constraints caused by scarce fault data. Qaid et al. [24] addressed the interpretability limitations of traditional deep learning by proposing the Fault Diagnosis Large Language Model (FDL-LLM) framework, which achieves dual objectives of fault classification and interpretable diagnosis through spectral data string encoding and instruction tuning, providing a comparative analysis with DiagLLM. Xiao et al. [148] introduced the Knowledge-Guided Semantic Representation Large Language Model (KG-SR-LLM) framework, transforming vibration time-series data into natural language semantic sequences and achieving a 98.36% average accuracy and 0.1162 s inference latency per sample via Low-Rank Adaptation (LoRA) lightweight fine-tuning, with a memory footprint of only 1142 MB, demonstrating edge deployment potential despite performance attenuation under extreme operating conditions. Jeong et al. [149] tackled the challenge of LLMs’ limited context windows for processing long-sequence high-dimensional signals by proposing an efficient input construction strategy combining feature enhancement, PCA dimensionality reduction, multi-strategy Retrieval-Augmented Generation (RAG), and prompt engineering, underscoring the importance of refined, high-quality inputs.

In the last 2 years, this direction has advanced rapidly, and several representative 2025–2026 studies extend LLM-assisted diagnosis toward acoustic and non-contact modalities. At the acoustic end, He et al. [23] converted AE features of full ceramic bearings into structured text for GPT-2 fine-tuning, achieving high accuracy with as few as five labeled samples. At the non-contact multimodal end, Lu et al. converted dynamic vision data into tokens for a multimodal LLM, demonstrating intelligent diagnosis without contact measurement [25]; a physics-informed multimodal large model with dynamic vision further provided expert-level textual explanations at accuracy comparable to contact vibration sensing [26]. In parallel, signal-to-text multimodal frameworks have matured rapidly: FR-LLM jointly performs fault diagnosis and remaining useful life prediction with frequency-domain textual prompts [27]; semantic-aware cross-modal alignment purifies sensor signals in a unified semantic space with SNR-based gating [28]; CoFAE-LLM fuses multi-view evidential reasoning into soft prompt embeddings [29]; HCMA_GPT aligns signal and text hierarchically to bridge the signal–text modality gap [30]; and a digital twin-powered large vision-language model augments scarce industrial samples for multi-scenario diagnosis [31]. A recent systematic review of 58 studies reports that multimodal foundation models raise out-of-distribution accuracy from 18.25% to 71.95% and achieve about 98% diagnostic accuracy with only 1.2% labeled samples, while retrieval-augmented generation and knowledge graph grounding are emerging as the principal remedies for hallucination [150]. It should be noted that, except for the acoustic emission study of He et al. [23], these frameworks operate on vibration or visual modalities; airborne acoustic LLM diagnosis remains largely unexplored, which further motivates the audio foundation model direction emphasized in this outlook. These advances substantiate the outlook of this review: LLM-assisted acoustic diagnosis should evolve from static signal-to-text fine-tuning toward physically grounded, retrieval-augmented, multimodal reasoning, with audio foundation models and non-contact sensing as the two enabling pillars while retaining its role as a trustworthy decision-support layer rather than a replacement for real-time edge diagnostic systems.

Nevertheless, inherent risks such as LLM hallucination [151], coupled with a deficient understanding of the physical mechanisms and causal relationships governing fault signals and an innate weakness in processing continuous numerical and temporal features, necessitate their collaborative operation with signal-processing-based deep-learning models. Before LLM-assisted diagnosis can be considered trustworthy for industrial deployment, six requirements should be addressed: (i) validation of model outputs against expert ground truth rather than against fluency alone; (ii) uncertainty calibration, so that a model reports confidence consistent with its actual reliability and abstains when evidence is insufficient; (iii) hallucination control through retrieval-augmented generation, knowledge graph grounding [150], and constrained decoding; (iv) computational latency, which excludes autoregressive LLMs from real-time edge loops and motivates the cloud-edge division discussed in Section 6.3; (v) resource requirements, quantified against the complexity contrast in Section 6.3; and (vi) physics-informed or knowledge-constrained designs, which anchor the reasoning to the propagation physics of Section 6.1.1 and to structured fault knowledge rather than to free-form generation. Future research should explore a hierarchical architecture that integrates real-time monitoring via signal-processing models with in-depth analysis and knowledge reasoning via LLMs, employing structured knowledge graphs to constrain LLM outputs, thereby suppressing hallucinations and elevating decision-making credibility.

### 6.3. Expansion of Non-Contact Monitoring Systems

Engineering deployment serves as the critical bridge connecting laboratory algorithms to industrial field applications and represents the prevailing trend in translating theoretical research into practical implementation. Existing studies predominantly rely on validation using standard laboratory datasets. However, complex industrial scenarios characterized by variable speeds and loads, intense background noise, multi-source acoustic aliasing, and diverse bearing specifications result in a significant performance gap between laboratory algorithms and practical applications.

Taking the few-shot and cross-domain generalization literature reviewed in Section 5.2 as an example, the dominant public benchmark remains the CWRU laboratory dataset. Although this dataset has driven rapid progress in algorithm development, it poses three fundamental limitations for acoustic diagnosis research: (i) modality mismatch: CWRU provides only contact-measured vibration accelerations and no airborne acoustic recordings, so generalization performance measured on it cannot reflect the acoustic transfer path (geometric spreading, atmospheric absorption, reverberation) analyzed in Section 6.1.1; (ii) environmental simplicity: laboratory acoustics with a single test rig and low ambient noise differ fundamentally from industrial scenes with multi-source aliasing and strong background noise; and (iii) fault-mode bias: seeded single-point defects cannot represent naturally evolved distributed faults. Open benchmark datasets that contain synchronized airborne acoustic bearing signals under industrial conditions are still scarce (Table 6); most acoustic studies therefore rely on self-collected data, which hampers fair comparison and reproducibility. Consequently, we recommend that future acoustic diagnosis studies adopt a two-tier validation protocol: fine-grained algorithm comparison on public laboratory benchmarks for reproducibility, plus field validation on actual industrial acoustic data with variable speed, load, and sensor distance to demonstrate engineering practicability. Cross-benchmark validation, extending from CWRU-like laboratory data to industrial acoustic field data, should become a standard requirement when reporting few-shot and cross-domain generalization results.

Addressing the challenge of generalization across variable and cross-operating conditions, Wang et al. [153] proposed a Multi-Level Feature Encoder (MLFE). This method combines frequency masking preprocessing for denoising with statistical feature extraction in an unsupervised manner, offering a novel solution for cross-domain acoustic signal fault diagnosis. Wang, D et al. [154] designed a Hybrid Attention-driven Convolutional Adversarial Network (HA-CAN), utilizing dense convolutions to enhance feature propagation and minimizing feature distribution discrepancies across different operating conditions via the Wasserstein distance. This approach achieved an average diagnostic accuracy exceeding 97% in two experimental sets, effectively mitigating the insufficiency of acoustic signal cross-domain generalization.

Regarding customized solutions for specific industrial scenarios, Huang et al. [152] addressed Doppler distortion and intense noise in wayside acoustic diagnosis for rail vehicle wheel bearings. They proposed a method combining a Uniform Rectangular Array (URA) with a Minimum Variance Distortion-less Response (MVDR) optimal spatial filter, successfully extracting fault features even under extreme noise conditions of −39.14 dB. Zhang et al. [155] tackled noise interference and sample scarcity in conveyor roller anomaly detection by implementing a multi-frame fused Mel-Frequency Cepstral Coefficients and Sample Center Distance-weighted Support Vector Data Description (SVDD) method. This approach improved accuracy from 79.67% (traditional single-frame SVDD) to 92.56%, and precision from 76.41% to 90.31%.

In terms of diagnostic systems and hardware development, De Pontes et al. [156] and Celebioglu et al. [157] independently proposed smartphone-based acoustic diagnostic solutions. By deploying lightweight models, they enabled low-cost portable detection, facilitating the transition from algorithms to commercial products. Furthermore, the expansion into non-contact monitoring modalities such as current and temperature offers additional pathways for engineering deployment. Jabbari et al. [158] utilized Wiener filtering and Empirical Wavelet Transform to process DC motor current signals, inputting time-frequency grayscale images into a CNN to accurately identify bearing faults and stator winding short circuits, marking the first application of drive DC current for motor fault diagnosis. Janssens et al. [159] combined Infrared Thermography (IRT) with machine learning to identify lubrication contamination by hard particles and shaft imbalance, overcoming the detection lag inherent in traditional temperature monitoring and providing a new scheme for non-contact incipient fault monitoring. Although these studies have facilitated the transition of acoustic signal diagnosis from laboratory settings to industrial sites, core obstacles to large-scale deployment persist. These include algorithmic stability under extreme operating conditions, adaptability to non-standard equipment, and the need for continuous optimization in data-scarce scenarios.

However, whether these advances in algorithms, customized solutions, and hardware can ultimately be deployed at the edge is determined by the computational and memory budgets of the diagnostic models themselves. Table 7 quantitatively contrasts the complexity of representative models discussed in this review.

Three observations follow from the contrast. First, lightweight designs reduce parameter counts and FLOPs by one to two orders of magnitude relative to standard Transformer backbones [63,126,137] while retaining competitive accuracy on bearing benchmarks, which is the prerequisite for deployment on resource-constrained edge devices such as industrial gateways and embedded boards. Second, the reported inference latencies of lightweight models already satisfy the real-time requirements of online acoustic monitoring, and their memory footprints, reduced to a few megabytes after int8 quantization, further enable on-device storage of multiple fault classes. Third, LLM-based approaches remain several orders of magnitude larger [148,149], which makes edge inference physically impractical; a cloud-edge collaborative architecture, in which lightweight models perform online screening at the edge and LLMs provide offline interpretation and decision support in the cloud, is therefore the recommended deployment topology. These quantitative contrasts demonstrate that the lightweight deployment route discussed in this review is not only feasible but also the only realistic path for edge-level real-time acoustic diagnosis.

In summary, the development of acoustic signal-based bearing fault diagnosis technology has entered a pivotal transitional phase, evolving from isolated algorithms to integrated systems and from laboratory settings to industrial applications. Future research endeavors must deepen the physical alignment and complementary mechanisms between acoustic and heterogeneous signals. Concurrently, it is imperative to clarify the auxiliary role of Large Language Models and optimize their lightweight deployment capabilities. Furthermore, through engineering-oriented packaging, laboratory algorithms must be transformed into reusable, industrial-grade solutions, with validation extended from laboratory benchmarks to industrial acoustic field data. This progression is essential to bridge the gap from feasibility verification to industrial-grade reliability, thereby providing core technological support for the full lifecycle health management of high-end equipment.

## 7. Conclusions

This paper presents a comprehensive review of bearing fault diagnosis methodologies utilizing vibration and acoustic signals. Adopting a holistic perspective that spans the entire pipeline from signal acquisition and feature extraction to intelligent diagnosis, it presents a detailed overview of specific diagnostic implementations leveraging machine-learning and deep-learning paradigms for both signal modalities. The study conducts a comparative analysis of the respective advantages and limitations inherent in vibration-based and acoustic-based measurement techniques. Furthermore, it traces the technical evolution from conventional manual feature engineering to contemporary end-to-end deep-learning diagnosis, highlighting innovative diagnostic model applications derived from the distinct physical characteristics of these two signal types. Finally, the paper posits that future research should prioritize the development of a multi-modal diagnostic architecture centered on the deep fusion of physics-informed and data-driven principles. It is imperative to delineate the auxiliary role of Large Language Models in fostering trustworthy and interpretable diagnosis while concurrently advancing lightweight deployment and robust cross-condition generalization capabilities. Moreover, heterogeneous modality alignment should be anchored on the shared kinematic fault characteristic frequencies, with propagation-delay compensation and envelope-domain alignment, to unlock the full potential of physically grounded multi-modal fusion. Such efforts are essential to construct a resilient, intelligent monitoring system applicable to full-scenario industrial environments.

## Figures and Tables

**Table 1:** Comparison of airborne acoustic, acoustic emission, and vibration measurements for bearing fault diagnosis.

Dimension	Airborne Acoustic	Acoustic Emission (AE)	Vibration
Propagation medium	Air	Solid (elastic waves)	Solid (mechanical waves)
Contact	Non-contact	Contact (surface-coupled)	Contact (mounted)
Typical bandwidth	20 Hz–20 kHz	100 kHz–1 MHz	Up to tens of kHz (typically 0–10 kHz)
Attenuation mechanisms	Geometric spreading, atmospheric absorption, reflection/reverberation	Material damping, path-dependent attenuation in solid	Multi-interface transmission, joint low-pass filtering, structural resonance amplification
Diagnostic advantages	Non-contact acquisition, flexible placement, array-based spatial localization, complementarity with vibration	High sensitivity to incipient micro-cracks and plastic deformation, early warning of weak signals	Mature methodology, rich benchmark support, well-established fault characteristic frequencies

**Table 2:** Noise conditions adopted in representative acoustic denoising studies for bearing fault diagnosis.

Study	Denoising Approach	Noise Type	SNR Levels Evaluated	Downstream Evaluation
Cong et al. [130]	Loudness-based feature extraction and adaptive filtering	Non-stationary ambient noise, airflow noise and multipath reverberation	Low SNR scenario, quantified by Fault Frequency Prominence	Rolling bearing outer race fault diagnosis via envelope spectrum
Wu et al. [131]	AVMD-IMVO-MCKD compound fault extraction	Gaussian white noise and industrial composite noise	−8 dB simulation, natural noisy experiment	Inner and outer race compound fault feature separation
Ding et al. [134]	Deep learning noise reduction (wind turbine)	Mixed realistic noise, white noise, mechanical noise, wind noise	Four levels, low, mild, moderate, severe	Five-type bearing fault classification with SVM and CNN
Wang et al. [63]	WAA-FMD and LGAF-Swin Transformer	Strong Gaussian white noise and industrial ambient noise	−13, −11, −9, −7, −5 dB (5 levels)	Rolling bearing fault classification with accuracy, F1, Kappa, and AUC
Luo et al. [136]	Enhanced feature extraction network	Gaussian white noise and ambient background noise	Clean signal and 2 dB noise condition	7-type bearing fault classification with accuracy and anti-noise performance

**Table 3:** Representative benchmark case studies of acoustic signal-based bearing fault diagnosis.

Study	Method	Benchmark Dataset	Task	Best Reported Accuracy
Cong et al. [130]	Loudness-based feature extraction and adaptive filtering	6206 rolling bearing outer race fault acoustic dataset	Impact feature extraction	Fault Frequency Prominence = 5.8
Wu et al. [131]	AVMD-IMVO-MCKD	Simulated compound fault signal, NU205 bearing acoustic dataset	Compound fault feature extraction	Inner and outer race fault frequency separated via envelope spectrum
Ding et al. [134]	Deep learning noise reduction (wind turbine)	Simulated realistic working conditions based on KAIST bearing dataset	Diagnosis under realistic noise	99.30% for CNN, 97.98% for SVM
Wang et al. [63]	WAA-FMD and LGAF-Swin Transformer	KAIST bearing acoustic dataset, self-built FAG 30205 bearing dataset	Diagnosis under strong noise	100% on KAIST set, 98.62% on self-built set
Luo et al. [136]	Enhanced feature extraction network	MB ER-8K bearing acoustic array dataset	Fault classification	98.52% under clean signal, 96.62% under 2 dB noise
Peng et al. [137]	Phoneme-inspired lightweight Transformer	Two bearing acoustic datasets	Fault classification	98.2 percent on BD dataset
He et al. [23]	GPT-2 few-shot learning	Full ceramic bearing acoustic emission lab dataset	Few-shot classification	92.34 percent at 5-way 5-shot
Altaf et al. [128]	Machine learning on sound features	Lab dataset MB ER-12K	Fault detection	99.1 percent with KLDA and combined bandpass features

**Table 4:** Experimental conditions of representative acoustic bearing fault diagnosis studies.

Study	Machine	Fault Type and Severity	Sensor Configuration	Recording Conditions	Environment
Cong et al. [130]	Bearing, 9 balls	0.2 × 0.5 mm defect	Capacitive microphone, distance NR	25.6 kHz, band < 12.8 kHz; 1200 rpm	Lab (controlled)
Wu et al. [131]	NU205 bearing, QPZZ-II bench	Compound faults (inner and outer ring)	2 microphones, perpendicular, 0.5 m	8.192 kHz; 13.33 Hz	Lab
Ding et al. [134]	NSK 6205 DDU (KAIST)	Machined cracks FI03/FI10/FO03/FO10	PCB 378B02 microphone	51.2→44.1 kHz; 3010 rpm; 0 Nm; 60 s records	Lab and field-recorded noise
Wang et al. [63]	NSK 6205, FAG 30205	Inner, outer, roller faults	PCB 378B02 microphone	51.2 kHz; 8192 points	Lab, noise to −13 dB
Luo et al. [136]	Rolling bearing	Rolling bearing	Mic array (ring disc)	1200 rpm; 1024–point samples	Lab
He et al. [23]	Full ceramic bearings	Inner/outer/ball/cage and normal (5 classes)	AE sensors (contact)	5 MHz; 600 rpm	Lab
Altaf et al. [128]	Rolling bearings, test rig	Seeded faults	Single microphone	Sound recording, non-contact; sampling rate NR	Lab

**Table 5:** Physical differences between vibration and airborne acoustic paths and their implications for fusion design.

Property	Vibration Path	Airborne Acoustic Path	Fusion Implication
Medium and speed	Solid, ≈$c_{solid}$ 5000 m/s	Air, $c_{air}$ ≈ 343 m/s	Acoustic stream lags by $\tau =d_{m}/c_{a}$, compensate before fusion
Carrier band	Structural resonance band	Broadband radiation (20 Hz–20 kHz)	Align at the shared $f_{c}$ in the envelope domain; carriers complementary
Attenuation	Joint low-pass filtering, multi-interface reflection	Spreading ∝ 1/r, absorption ∝ f^2^, reverberation	Reliability varies with distance; use as fusion confidence weight
Interference sensitivity	Insensitive to airborne noise; mounting-dependent	Sensitive to background noise, multi-source aliasing	Cross-modal redundancy suppresses blind spots
Contact	Contact mounting	Non-contact, flexible placement	Monitors where mounting is infeasible

**Table 6:** Comparison of widely used bearing fault datasets and their limitations for acoustic generalization studies.

Dataset	Modality	Environment and Fault Type	Limitations for Acoustic Studies
CWRU	Vibration	Lab rig; EDM point defects	No acoustic channel; artificial faults
MFPT	Vibration	Lab rig; seeded and real faults	No acoustic channel; limited conditions
XJTU-SY	Vibration	Lab life test; natural faults	No acoustic channel; no industrial noise
Paderborn	Vibration, cur-rent	Modular rig; real and seeded	No acoustic channel
IMS (NASA)	Vibration	Lab run-to-failure; natural	No acoustic channel; outdated acquisition (2003–2004, 20 kHz)
Self-collected acoustic [152]	Airborne acoustic	Field; real faults	Not standardized; limited comparability

**Table 7:** Computational complexity and memory consumption of representative models for bearing fault diagnosis and their implications for edge deployment.

Model (Category)	Params (M)	FLOPs (G)	Size, FP32 (MB)	Ref.
ResNet-18 (CNN)	11.7	1.8	46.8	[160]
MobileNetV2 (lightweight CNN)	3.4	0.3	13.6	[161]
Swin-T (Transformer)	28.3	4.5	113	[162]
LLM-based diagnosis: DiagLLM	7000	Not reported	28,000	[147]
GPT-2 small	124	per token	496 (GPT-2 small)	[23]

## Data Availability

No new data were created or analyzed in this study. Data sharing is not applicable to this article.
